# Entropy-Fused Enhanced Symplectic Geometric Mode Decomposition for Hybrid Power Quality Disturbance Recognition

**DOI:** 10.3390/e27090920

**Published:** 2025-08-30

**Authors:** Chencheng He, Wenbo Wang, Xuezhuang E, Hao Yuan, Yuyi Lu

**Affiliations:** College of Science, Wuhan University of Science and Technology, Wuhan 430065, China

**Keywords:** PQD, improved symplectic geometric mode decomposition, refined generalized multiscale quantum entropy, refined generalized multiscale reverse dispersion entropy, double-layer deep extreme learning machine

## Abstract

Electrical networks face operational challenges from power quality-affecting disturbances. Since disturbance signatures directly affect classifier performance, optimized feature selection becomes critical for accurate power quality assessment. The pursuit of robust feature extraction inevitably constrains the dimensionality of the discriminative feature set, but the complexity of the recognition model will be increased and the recognition speed will be reduced if the feature vector dimension is too high. Building upon the aforementioned requirements, in this paper, we propose a feature extraction framework that combines improved symplectic geometric mode decomposition, refined generalized multiscale quantum entropy, and refined generalized multiscale reverse dispersion entropy. Firstly, based on the intrinsic properties of power quality disturbance (PQD) signals, the embedding dimension of symplectic geometric mode decomposition and the adaptive mode component screening method are improved, and the PQD signal undergoes tri-band decomposition via improved symplectic geometric mode decomposition (ISGMD), yielding distinct high-frequency, medium-frequency, and low-frequency components. Secondly, utilizing the enhanced symplectic geometric mode decomposition as a foundation, the perturbation features are extracted by the combination of refined generalized multiscale quantum entropy and refined generalized multiscale reverse dispersion entropy to construct high-precision and low-dimensional feature vectors. Finally, a double-layer composite power quality disturbance model is constructed by a deep extreme learning machine algorithm to identify power quality disturbance signals. After analysis and comparison, the proposed method is found to be effective even in a strong noise environment with a single interference, and the average recognition accuracy across different noise environments is 97.3%. Under the complex conditions involving multiple types of mixed perturbations, the average recognition accuracy is maintained above 96%. Compared with the existing CNN + LSTM method, the recognition accuracy of the proposed method is improved by 3.7%. In addition, its recognition accuracy in scenarios with small data samples is significantly better than that of traditional methods, such as single CNN models and LSTM models. The experimental results show that the proposed strategy can accurately classify and identify various power quality interferences and that it is better than traditional methods in terms of classification accuracy and robustness. The experimental results of the simulation and measured data show that the combined feature extraction methodology reliably extracts discriminative feature vectors from PQD. The double-layer combined classification model can further enhance the model’s recognition capabilities. This method has high accuracy and certain noise resistance. In the 30 dB white noise environment, the average classification accuracy of the model is 99.10% for the simulation database containing 63 PQD types. Meanwhile, for the test data based on a hardware platform, the average accuracy is 99.03%, and the approach’s dependability is further evidenced by rigorous validation experiments.

## 1. Introduction

High-power nonlinear and impact loads significantly degrade power quality, jeopardizing the stability and integrity of power supply systems [1]. Effective power quality management relies on the accurate identification and classification of disturbance signals [2], which forms the basis for targeted improvements in power supply quality [3,4]. Existing methodologies for detecting and identifying such disturbances have been extensively reviewed in the literature [5], highlighting practical and efficient identification schemes [6]. Key signal processing techniques include STFT, S-transform, WT, EMD, VMD, and Gabor–Wigner transform [7]. The results show that STFT has a modal aliasing rate of more than 40% in non-stationary signal analysis [8]. Energy leakage from wavelet transforms results in a feature redundancy of up to 60% [9]. The IMF correlation coefficient of EMD may be lower than 0.3 in the case of mixed disturbance and strong noise [10]. Recent innovations include the use of Gramian Angular Fields (GAFs) to transform perturbation signals into two-dimensional formats for enhanced feature extraction [11], as well as singular value decomposition for preprocessing transient perturbations [12].

Feature extraction is pivotal in perturbation recognition, as it directly influences the speed and accuracy of disturbance identification. Global research efforts have focused on developing diverse feature extraction methods [13]. For instance, a combination of S-transform and Hilbert–Huang transform, along with statistical methods (e.g., maximum difference, mean, standard deviation, and root mean square), has been used to extract 61 feature vectors from primary datasets [14]. Spectral kurtosis methodologies have also been employed for feature vector generation based on mathematical statistics [15]. Recent advancements in entropy-based concepts have led to the development of compact, discriminative feature vectors with minimal redundancy [16]. Energy entropy, for instance, has been widely adopted for eigenvector construction and dimensionality reduction [17]. Other techniques, such as multiscale entropy, fuzzy entropy, Kolmogorov entropy [18], and second-order Rényi entropy [19], have also been successfully applied to datasets processed via empirical mode decomposition [20], consistently demonstrating their effectiveness in distinguishing composite power quality disturbances.

To address the challenge of excessive feature dimensionality, researchers have introduced optimization algorithms, including genetic algorithms (GAs) [21] and random forest (RF) techniques [22]. For example, a Wrapper-style approach using RF reduced a 64-dimensional perturbation to 30 dimensions while enhancing feature discrimination [23]. Similarly, a multi-granularity space-based algorithm was developed to identify optimal features within a 60-dimensional perturbation [24]. However, traditional statistical methods often result in high-dimensional feature vectors, necessitating optimization algorithms to reduce complexity.

The rapid advancement of artificial intelligence has facilitated the widespread adoption of neural network models for power quality disturbance recognition, owing to their ability to process large datasets [25]. Recent innovations include the direct adoption of one-dimensional perturbation data as input to CNN frameworks [26], the application of Deep Belief Networks (DBNs) for improved classification accuracy [27], and the design of hybrid models integrating Bi-directional Long Short-Term Memory (BiLSTM) with attention mechanisms to capture temporal dependencies [28]. Additionally, GANs have been applied in data augmentation, addressing the issue of insufficient training data [29]. Despite their performance, deep learning methods face challenges such as structural complexity, training difficulties, and variability in feature extraction effectiveness, limiting their stability and universality.

From the above analysis, it is evident that existing methods for power quality disturbance feature extraction often struggle to balance feature effectiveness, recognition accuracy, and complexity control, particularly when processing complex disturbance signals. To overcome the limitations of conventional approaches, this study develops a multi-dimensional feature extraction framework based on RGMQE and RGMRDE, building upon the ISGMD framework. This approach is used to reduce the dimensionality and complexity of feature vector extraction while maintaining feature effectiveness and recognition accuracy. This study presents three novel contributions, which are outlined below.

- According to the characteristics of power quality disturbance, the embedding dimension and component adaptive screening method of symplectic geometric mode decomposition are improved. The embedding dimension for symplectic geometric mode decomposition is calculated by phase space reconstruction, the Pearson correlation coefficient is employed for adaptive filtering and reconstruction of the modal components. The stability and adaptability of feature extraction are enhanced by the improved symplectic mode decomposition to preprocess the disturbed signal.
- Using an improved Sinusoidal Geometric Modal Decomposition method, the power quality disturbance signal is decomposed into three components: low-frequency disturbance, intermediate-frequency disturbance, and high-frequency disturbance. The disturbance features are combined with refined generalized multiscale quantum entropy and refined generalized multiscale quantum entropy to construct high-precision and low-dimensional feature vectors.
- A double-layer deep extreme learning machine (DLDELM) network was constructed to identify and verify the disturbed signal. The simulation power quality disturbance signal is generated by Matlab2022a/Simulink, and the measured power quality disturbance signal is generated by the disturbance identification experiment platform built. According to the suggested approach, the features of disturbance signals are extracted, and the disturbance type is identified by the DLDELM. The experimental outcomes demonstrate that the proposed approach exhibits high recognition precision, robustness against noise, and scalability.

## 2. Model Decomposition Analysis

### 2.1. Symplectic Geometric Mode Decomposition

The core of symplectic geometric mode decomposition lies in the structure-preserving properties on symplectic manifolds, thereby obtaining the corresponding symplectic geometric components.

Suppose we have a signal $x(t)=x_{1},x_{2},\ldots x_{n}$. According to Takens’ [30] embedding theorem, the projection x of the signal onto the embedding matrix X is as follows:

(1) $$X=\left[\begin{array}{c} \begin{array}{cccc} x_{1} & x_{1+t} & \ldots & x_{1+(e-1)t} \end{array}\\ \begin{array}{cccc} \vdots & \vdots & \vdots & \vdots \end{array}\\ \begin{array}{cccc} x_{p} & x_{p+t} & \ldots & x_{p+(e-1)t} \end{array} \end{array}\right]$$

where e is the embedded dimension; p=q−(e−1); t is the delay time. The C-C algorithm is used for the determination of τ [31]. Autocorrelation analysis was performed on X, thus obtaining the covariance symmetric matrix A:

(2) $$A=X^{T}X$$

A Hamiltonian matrix W is constructed:

(3) $$W=\left[\begin{array}{cc} A & 0\\ 0 & -A^{T} \end{array}\right]$$

$N=W^{2}$ is the Hamiltonian matrix. Then a symplectic orthogonal matrix Q is constructed.

(4) $$Q^{T}NQ=\left[\begin{array}{cc} B & R\\ 0 & B^{T} \end{array}\right]$$

where R is the sub-matrix after the matrix transform; Matrix B takes the form of an upper triangular matrix. The characteristic values are the following: $\lambda_{1},\lambda_{2},\lambda_{3},\ldots ,\lambda_{D}$. The eigenvalue of A is as follows:

(5) $$\xi_{i}=\sqrt{\lambda_{i}}(i=1,2,\ldots ,e),\xi_{1}>\xi_{2}>\ldots >\xi_{e}$$

where the distribution of $\xi_{i}$ is the symplectic geometry spectrum of A, $Q_{i}(i=1,2,\ldots e)$ is a feature vector of $\xi_{i}$, and the steps of the reconstruction of each component matrix are as follows.

First, the matrix of transformation coefficients is calculated:

(6) $$S_{i}=Q_{i}^{T}X^{T}$$

Then, transform $S_{i}$ to obtain a single-component $Y_{i}$

(7) $$Y_{i}=Q_{i}S_{i}$$

where i=1,2,…e. The initial trajectory matrix Y for single-component signals was then constructed:

(8) $$Y=Y_{1}+Y_{2}+\ldots Y_{\theta }$$

where $Y\in R^{\omega \times \theta }$. Define the element in $Y_{i}$ as $Y_{ij}$, 1≤i≤θ, 1≤j≤ω and $\theta^{*}=\min(\omega ,\theta ),\;\omega^{*}=\max(\omega ,\theta ),\;q=\omega +(\theta -1)t$.

(9) $$y_{ij}^{*}=\left\{\begin{array}{c} y_{ij},\;\omega <\theta \\ y_{ji},\;\omega \geq \theta \end{array}\right.$$

Subsequently, by applying the diagonal averaging operation, the transform matrix is given by the following:

(10) $$E_{k}=\left\{\begin{array}{c} \frac{1}{\phi }\sum_{\delta =1}^{\phi }y_{\delta ,\phi -p+1}^{*},\;1\leq \phi \leq \theta^{*}\\ \frac{1}{\theta^{*}}\sum_{\delta =1}^{\theta^{*}}y_{\delta ,\phi -p+1}^{*},\;\theta^{*}<\phi \leq \omega^{*}\\ \frac{1}{q-\phi +1}\sum_{\delta =\phi -m^{*}+1}^{q-\omega^{*}+1}y_{\delta ,\phi -p+1}^{*},\;\omega^{*}<\phi \leq q \end{array}\right.$$

Diagonally averaging transforms a matrix Y into a matrix E of q×θ dimensions. Thus, the signal x is decomposed into q Initial Symplectic Geometric Mode Components (ISGMCs) with different trend terms and different frequency bands are as follows:

(11) $$E=E_{1}+E_{2}+\ldots +E_{d}$$

### 2.2. Improved Symplectic Geometric Mode Decomposition

#### 2.2.1. The Determination of the Number of Embedding Dimensions

The selection of embedding dimension critically determines the signal decomposition outcomes. However, the energy spectral density method is used through a false nearest neighbor analysis of the signal with a difference of three orders of magnitude between the main frequency and the sampling frequency. To solve this problem, the Cao algorithm [32] provides a solution. This algorithm determines the appropriate embedding dimension by calculating a(i,d), which is mainly used to measure the change in proximal state vectors in reconstructed phase space. Since the characteristics of the signal at different phase points i may be significantly different (for example, some regions change sharply, while some regions change gently), the threshold needs to be dynamically adjusted for different phase points to adapt to the local characteristics of the signal to guarantee algorithmic robustness and measurement accuracy.

Specifically, the Cao algorithm proposed in the literature takes the stop change in $E_{1}(d)$ as the standard to determine the embedding dimension, and its threshold is determined by the derivative of the underlying signal. For different phase points, the threshold should in principle have different values. In addition, different time series data may have different thresholds.t. This means that it is difficult or even impossible to give a universal threshold independent of dimension r, locus points, and specific time series data. Therefore, the setting of the threshold needs to be dynamically adjusted according to the local characteristics of the signal. To avoid the above problems, we define E(r) as shown in Formula (12).

(12) $$E(r)=\frac{1}{L-r\varsigma }\sum_{\delta =1}^{L-r\varsigma }\beta (\delta ,r)$$

where E(r) only depends on dimension r and lag ς. Its change is studied from r to r+1.

(13) $$E_{1}(r)=E(r+1)/E(r)$$

We found that $E_{1}(r)$ exceeds a specific threshold when r stops changing; If the time series originates from a phase point, then $r_{0}+1$ is the minimum embedding dimension we are looking for.

In fact, $E_{1}(r)$ is approaching a steady state, and it is difficult to determine the embedding dimension by observation alone. Based on this, a convergence criterion for the Cao algorithm is proposed. This criterion aims to enhance both the efficiency and precision of the algorithm. The steps are as follows:

- Define $\Delta_{i}=\left|\frac{E_{1}(i+1)}{E_{1}(i)}-1\right|,\;1\leq i\leq n-1$.
- Set a fluctuation threshold ζ of $E_{1}$. Iterate $E_{1}$ to find the first corresponding subscript of $\Delta_{i}<\zeta$, which is written as p.
- Define $\left\{\begin{array}{c} m_{r\varphi 1}=\frac{1}{2}(\Delta_{j}+\Delta_{\varphi +1})\\ m_{r\varphi 2}=\frac{1}{2}(\Delta_{\varphi +1}+\Delta_{\varphi +2})\\ m_{t\varphi }=\frac{1}{3}(\Delta_{\varphi }+\Delta_{\varphi +1}+\Delta_{\varphi +2}) \end{array}\right.$.
- Find the first subscript φ, which satisfies $(m_{r\varphi 1}-m_{t\varphi })(m_{r\varphi 2}-m_{t\varphi })<0$. When the embedding dimension is φ+1, $\Delta_{\varphi }>\Delta_{\varphi +1}>\Delta_{\varphi +2},\;p\leq \beta \varphi \leq n-2$.

#### 2.2.2. Adaptive Filtering of Components

For component reconstruction evaluation, Refs. [33,34] used periodic similarity and cosine similarity as metrics. Periodic similarity ensures comparable periods, while cosine similarity guarantees directional similarity. These limitations, however, can be overcome by applying the Pearson correlation coefficient (PCC).

Therefore, in order to address the issue of over-decomposition in symplectic geometric mode decomposition, this paper makes use of the Pearson correlation coefficient (PCC) to further optimize the decomposition results. Specifically, components are screened based on the PCC [35]. The PCC quantifies the degree of linear correlation between signals $x_{1}(t)$ and $x_{2}(t)$ by computing the covariance and standard deviation. Thus, after the decomposition is completed, a number of components can be filtered according to the correlation coefficient and a predefined threshold. Subsequently, components with strong correlations and high validity are retained, while those that are insignificant or have low correlations are excluded. The formula $\Psi_{1}$ for calculating the PCC of the first component is

(14) $$\Psi_{1}=\frac{\sum_{t=0}^{H-1}x_{1}(t)x_{2}(t)}{\sqrt{\sum_{t=0}^{H-1}{x_{1}}^{2}(t)\sum_{t=0}^{H-1}{x_{2}}^{2}(t)}}$$

where H corresponds to the temporal resolution of the sampled signal. Generally, when the PCC exceeds a value of 0.8, it indicates a strong linear correlation between two components. However, through experimental analysis, the correlation coefficient threshold used in this paper is set at 0.9.

### 2.3. Performance Analysis of Improved Symplectic Geometric Mode Decomposition

This paper evaluates the performance of ISGMD using quantified indicators, including calculation time, Intrinsic Mode Function (IMF) components, the Index of Overall Orthogonality (IOO), and the Index of Completeness (IC). The IOO and IC quantify the signal’s orthogonality and completeness, respectively, and describe the degree of modal aliasing and decomposition accuracy.

(15) $$I_{OO}=\frac{1}{A(A-1)}\sum_{a_{i},a_{j}=1}^{A}\sum_{t=0}^{T-1}\frac{I_{ai}(t)I_{aj}(t)}{I_{ai}(t)I_{aj}(t)}$$

(16) $$I_{CC}=\sqrt{\frac{\sum_{t=0}^{T-1}[x(t)-{\sum_{a=1}^{A}I_{a}(t)]}^{2}}{T-1}}$$

where $I_{a}(t)$ is the first component after decomposition a=1,2,…A, and A is the number of components $a_{i}\neq a_{j},\;a_{i}\in a,\;a_{j}\in a$.

**Figure 1:**
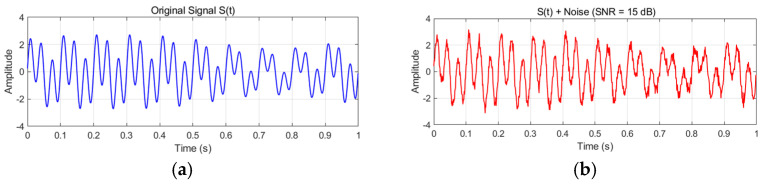
Analog signal waveform: (**a**) original signal waveform diagram; (**b**) waveform diagram of noisy signal.

The constructed analog signal, defined by Equation (17) and depicted in Figure 1, was decomposed using improved symplectic geometric mode decomposition (ISGMD), and its performance was analyzed. The algorithm’s performance metrics are summarized in Table 1, and Figure 1 displays the time domain depiction of the symplectic geometric elements.

(17) $$\left\{\begin{array}{c} S(t)=s_{1}(t)+s_{2}(t)+s_{3}(t)\\ s_{1}(t)=2\sin(1+0.5\sin(2\pi t))\sin(60\pi t)\\ s_{2}(t)=\sin(20\pi t)\\ s_{3}(t)=0.25\cos(20\pi t) \end{array}\right.$$

Based on Equation (17), we added 15 dB of Gaussian white noise. Figure 1 shows the original signal and the noise waveform.

**Figure 2:**
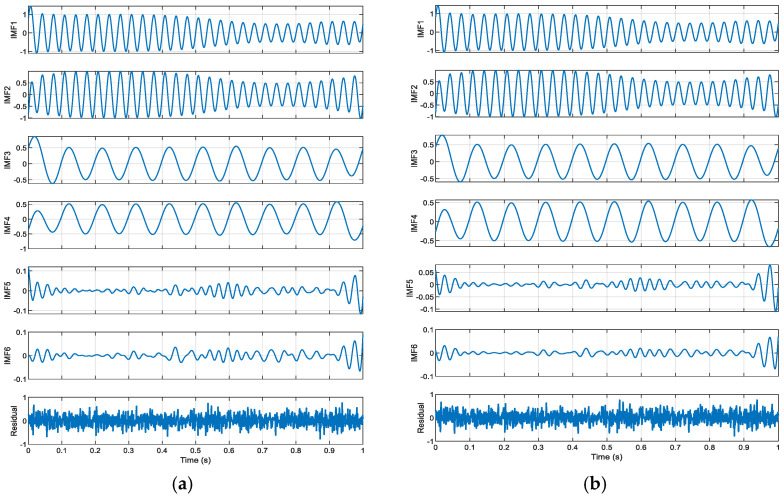
Time domain diagram of components of SGMD and ISGMD: (**a**) SGMD decomposition results; (**b**) ISGMD (α=0.4) decomposition results.

As shown in Figure 2, the residuals from SGMD exhibit distinct noise characteristics, while those from ISGMD are relatively smoother. By comparing the decomposition results of SGMD and ISGMD, it is evident that the two methods exhibit significant differences in the shape and amplitude of the IMF, indicating that the choice of decomposition method directly impacts the signal representation results. Among these, ISGMD provides capabi-ity through the α parameter (0.4), enabling more precise or accurate signal decomposition. This is particularly evident in the quality of the residuals—the residuals from ISGMD are smoother than those from SGMD, indicating that the improved algorithm performs better in terms of signal reconstruction integrity, highlighting the value of parameter optimization in enhancing decomposition accuracy and suppressing noise.

The adjustable parameters of ISGMD provide flexibility for specific signal processing requirements, and comparisons show that ISGMD has advantages in suppressing noise and improving decomposition accuracy. Additionally, 25 dB Gaussian white noise was added to this text for comparison. Below are the (α=0.3) decomposition results.

**Figure 3:**
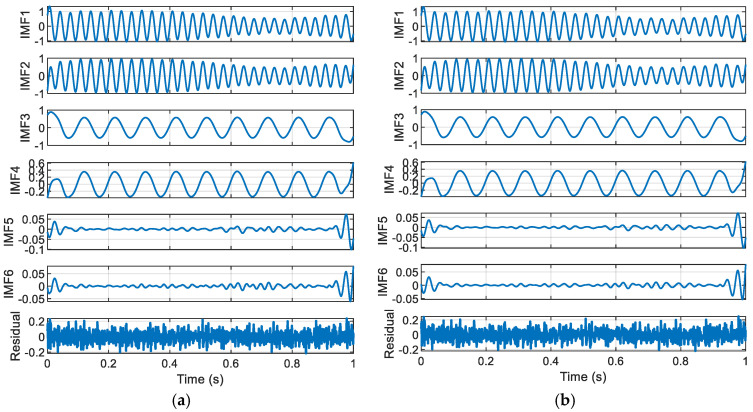
Time domain diagram of components of SGMD and ISGMD: (**a**) SGMD decomposition results; (**b**) ISGMD (α=0.3) decomposition results.

Figure 3 shows that the IMF components of SGMD may be more concentrated in fr-quency distribution, while the components of ISGMD may be more evenly distributed; the residuals obtained by the two methods differ in shape. SGMD is suitable for scenarios with high computational efficiency requirements and relatively simple signals; ISGMD is suitable for scenarios with high decomposition accuracy requirements and complex signals that require precise analysis.

The correlation coefficient for each disturbance component of 19 power quality disturbance types is calculated using Equation (2). We added the mathematical models of D1–D19 power mass disturbance signals, as shown in Table 2.

In Matlab2022a the threshold is set to 0.95, differing from the 0.2 threshold in [35]. We conducted a PCC threshold sensitivity analysis to discuss the impact of threshold selection on the classification results. We visualized the trends in sensitivity and specificity at different thresholds; sought a balance point where both sensitivity and specificity were high; considered the performance of accuracy and F1 scores; and determined the optimal threshold based on the intersection point or peak region of the curve. The impact of threshold selection on the classification results is shown in the figure below.

**Figure 4:**
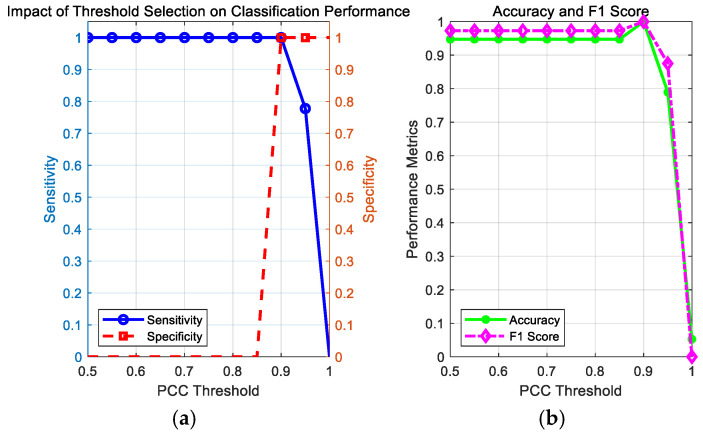
The impact of PCC threshold selection on classification model performance: (**a**) the impact of threshold selection on classification performance; (**b**) curves showing accuracy and F1 scores as a function of threshold.

Figure 4 illustrates the significant impact of PCC threshold selection on classifier performance. Figure 4a shows that the sensitivity curve remains near-perfect (>0.9) until the threshold approaches 1.0, after which it drops sharply, while the specificity curve remains at a low level (<0.2) before the threshold approaches 1.0, then sharply rises. This pattern of change reflects the classic trade-off between sensitivity and specificity, where increasing the threshold increases specificity but decreases sensitivity. Figure 4b further reveals that the two metrics reach an optimal balance point at a threshold of approximately 0.9, where the F1 score more sensitively reflects the trade-off characteristics between sensitivity and specificity compared to accuracy. Comprehensive analysis indicates that the optimal threshold is approximately 0.9, where the best overall performance can be achieved near this point. This provides important visual evidence and decision support for selecting an appropriate threshold in practical applications.

Table 3 shows the Pearson correlation coefficients of each component after signal decomposition.

As shown in Table 3, Imf 4 and Imf 5 are effective components for all perturbations. For single and composite disturbances involving harmonics, components from Imf 3 to Imf 5 are effective. For disturbances with oscillations and pulses, the effective components range from Imf 1 to Imf 5.

Based on Ref. [17] and Pearson correlation coefficient (PCC) calculations, the Imf corresponding to the bold-marked data in Table 3 is selected as the final ISGMD result.

## 3. Feature Extraction Based on Multiscale Entropy Reconstruction

Due to the diversity of disturbance signals, the distribution and characteristic manifestations of disturbance features vary across frequency bands. This variability leads to differences in the number of effective modes filtered by enhanced symplectic geometric decomposition. Moreover, the differences in feature manifestations result in diverse feature expressions, making feature extraction based on a single index insufficient.

To overcome the limitations of existing approaches, this study develops a multi-modal feature extraction architecture which combines the reconstruction of generalized composite multiscale quantum entropy and inverse dispersion entropy. This method qualitatively classifies different disturbance features by analyzing the frequency domain distribution and representation of power quality disturbance signals. It then extracts various types of disturbance features by computing the entropy of different functions, thereby obtaining high-precision, low-dimensional feature vectors.

### 3.1. Frequency Domain Analysis of Perturbation Signals

Following the IEEE Std1159-2019 power quality testing standard [36] and the relevant literature, a disturbance signal model was established. In the MATLAB 2022a environment, power quality disturbance, along with its signal model and parameter settings, was utilized for subsequent disturbance feature detection and classification to verify the performance of the relevant analysis algorithms.

The fundamental frequency of the perturbation model studied in this paper is 50 Hz, with a period of 0.02 s. During the simulation, the experimental configuration adopted a 10 kHz sampling frequency, and the sampling interval was 0.2 s.

#### 3.1.1. Low-Frequency Disturbances

By analyzing and defining the mathematical models of swell, sag, interrupt, and voltage flicker, we observe that the changes in these four disturbance models are primarily manifested in the fundamental frequency band. The disturbance signal is mainly characterized by amplitude variations in the 50 Hz frequency band.

To enhance the visibility of low-frequency components, 2 Hz and 5 Hz sine wave components are added to all signals; the sampling frequency is increased from 500 Hz to 1000 Hz to improve frequency resolution; and the signal time is extended from 0.4 s to 1.0 s to further improve frequency resolution. The Kaiser window is used instead of the Hanning window, which is more suitable for low-frequency analysis.

**Figure 5:**
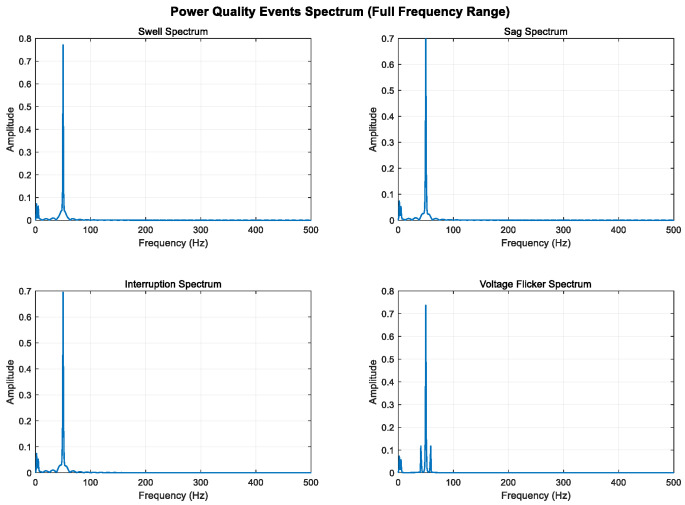
Full-band display of low-frequency components (0–500 Hz).

**Figure 6:**
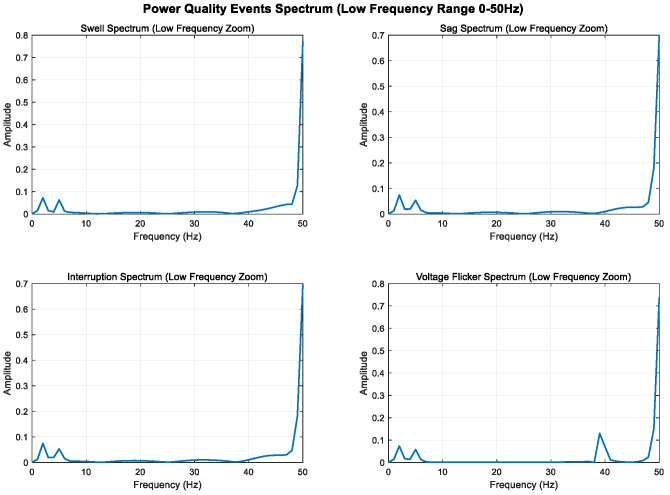
Local magnification of low-frequency band (0–50 Hz).

**Figure 7:**
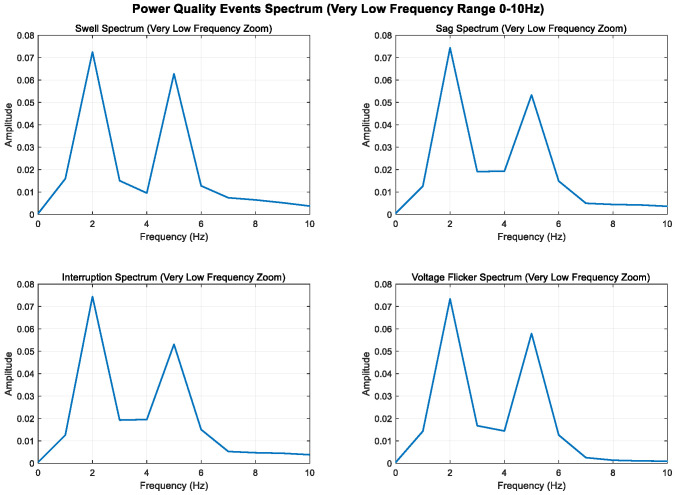
Local amplification of ultra-low-frequency band (0–10 Hz).

As shown in Figure 5, Figure 6 and Figure 7, higher sampling rates and longer time windows provide better frequency resolution (1000 Hz/1000 = 1 Hz); these modifications make the low-frequency components (2 Hz and 5 Hz) clearly visible in the spectrum diagram while maintaining the integrity of the original power quality event characteristics.

#### 3.1.2. Medium-Frequency Disturbances

Spectral leakage can be effectively reduced by combining Fourier analysis with windowing techniques. Window functions gradually attenuate the signal to zero in the time domain, preventing signal discontinuities; in the frequency domain, the window function’s spectrum is convolved with the original signal’s spectrum, concentrating the leaked energy near the main lobe and reducing side lobe interference.

To enhance the visibility of the mid-frequency components, sine wave components at 350 Hz and 650 Hz are added to all signals; the sampling frequency is increased from 1000 Hz to 3000 Hz to analyze frequency components up to 1500 Hz; the signal duration is extended from 0.4 s to 0.8 s to improve frequency resolution; and the Hanning window is replaced with the Blackman–Harris window to provide better spectral leakage suppression.

**Figure 8:**
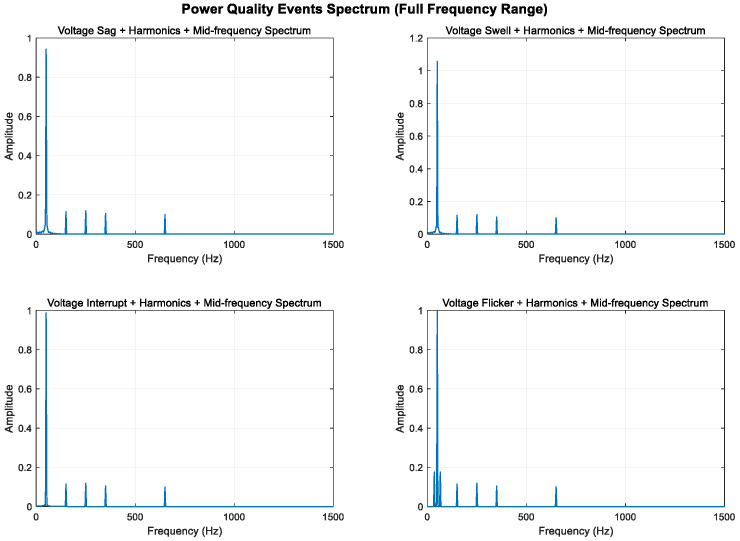
Full frequency range display of mid-frequency components (0–1500 Hz).

**Figure 9:**
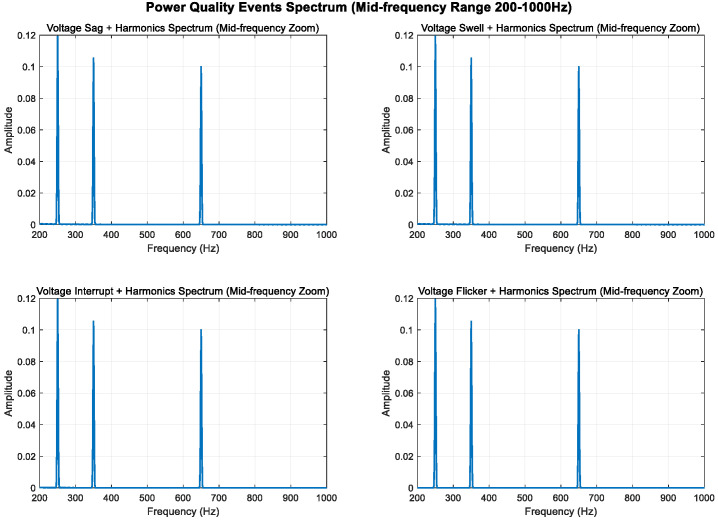
Mid-frequency local amplification (200–1000 Hz).

**Figure 10:**
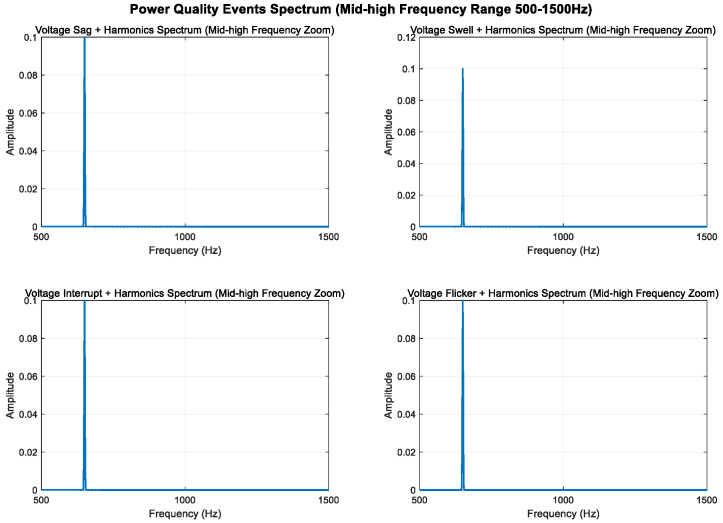
Localized amplification of mid–high frequency range (500–1500 Hz).

As shown in Figure 8, Figure 9 and Figure 10, higher sampling rates and longer time windows provide better frequency resolution (3000 Hz/2400 ≈ 1.25 Hz). These modifications make the mid-frequency components (350 Hz and 650 Hz) clearly visible in the spectrum diagram while maintaining the integrity of the original power quality components and harmonic characteristics.

#### 3.1.3. High-Frequency Disturbances

According to the mathematical model, voltage oscillation is a high-frequency signal that undergoes additional attenuation during disturbance. It is characterized by its distribution near the oscillation frequency band and amplitude changes within this band. In contrast, voltage pulses are formed by superimposing multiple AC and DC components onto a normal signal, resulting in a wide-spectrum signal with frequencies ranging from extremely low to extremely high [37].

An analysis of the mathematical models shows that the composite perturbations of oscillation, pulse, and harmonic retain their respective characteristics. To highlight more high-frequency components, the sampling frequency was increased from 3000 Hz to 10,000 Hz, enabling an analysis of frequency components up to 5000 Hz. High-frequency sine components at 1500 Hz and 2500 Hz were added, increasing the transient oscillation frequency to 1500 Hz. The higher sampling rate provides better frequency resolution.

**Figure 11:**
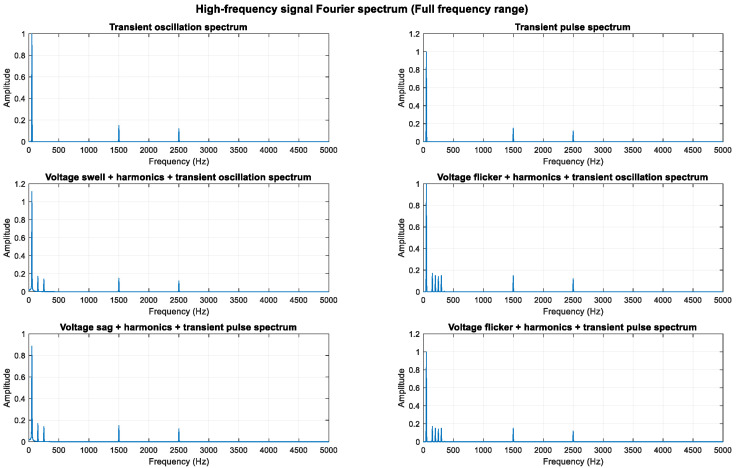
Full frequency band display of high-frequency components.

**Figure 12:**
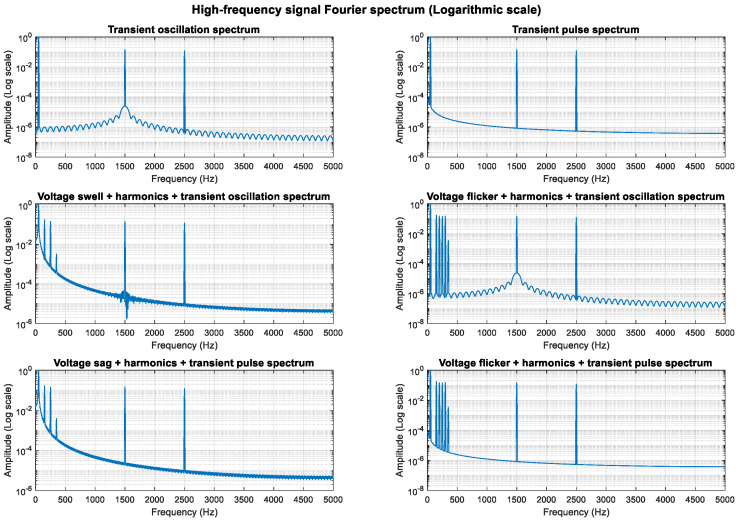
Full-band logarithmic scale display (enhanced small signal visibility).

**Figure 13:**
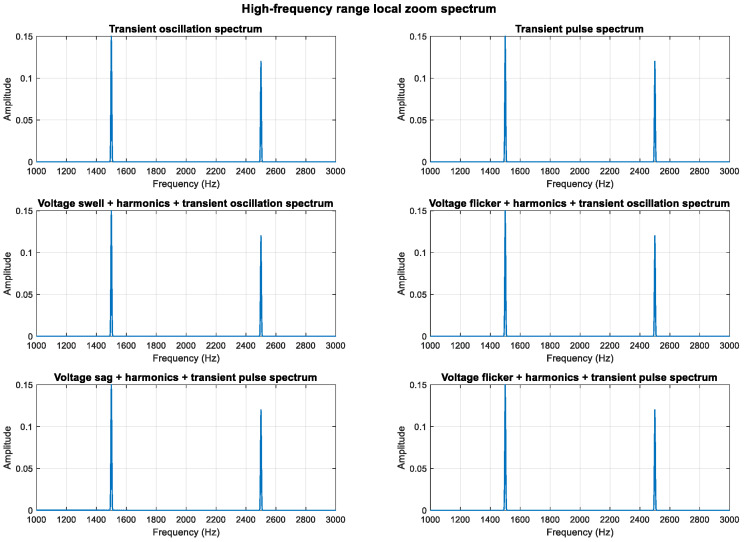
Localized magnification display in high frequency range.

As shown in Figure 11, Figure 12 and Figure 13, the high-frequency components (1.5 kHz and 2.5 kHz) are clearly visible in the spectrum diagram while maintaining the integrity of the original harmonic and transient characteristics.

### 3.2. Feature Extraction

Considering the complexity of power quality disturbances, which encompass a vast array of types, and the diverse manifestations of their features, such as amplitude-related and frequency-related characteristics, this paper introduces an advanced multi-domain feature extraction framework for PQD. This method relies on combinatorial reconstruction, aiming to achieve two goals simultaneously: reducing the feature dimension and ensuring the accuracy of feature extraction.

#### 3.2.1. Refined Generalized Multiscale Quantum Entropy

##### Multiscale Quantum Entropy

In the calculation of quantum entropy, the multifaceted nature of time series across various scales is often overlooked. To tackle this problem, researchers [38] have put forward the concept of multiscale quantum entropy which is grounded in quantum entropy (QE). The specific algorithmic procedure is as follows:

- Let the N dot scatter time series be Z={z(ε),1≤ε≤N}. The formula for calculating the coarse-grained vector is as follows:
  (18) $$y_{\omega }(\varepsilon )=\frac{1}{\omega }\sum_{\varepsilon =(\gamma -1)\omega +1}^{\gamma \omega }z(\varepsilon ),1\leq \gamma \leq N/\omega$$
  where ω represents the scale factor, ω=1,2,…,m, $m\in N^{*}$.
- The quantum entropy of the coarse-grained vector is computed and reconstructed. The formula for multiscale quantum entropy is derived as presented in Equation (19).
  (19) $$E_{MQE}(z,\omega ,m,\lambda )=E_{QE}(y_{\gamma }^{\omega },m,\lambda )$$
  where similar tolerance is r=(0.1~0.25)Q, where Q denotes the population standard deviation of the source sequence z(i).

##### Generalized Multiscale Quantum Entropy

To enhance the algorithm’s stability, this study generalizes the multiscale quantum entropy (MQE) framework by replacing coarse-grained mean computation with variance estimation across temporal scales. We introduce a generalized multiscale quantum entropy (GMQE) framework that extends traditional MQE through the following specific details:

- The reconstructed coarse-grained vector of Z={z(ε),1≤ε≤N} is as follows:
  (20) $$y_{g}^{\omega }(\gamma )=\frac{1}{\omega }\sum_{\varepsilon =(\gamma -1)\omega +1}^{\gamma \tau }{\left(z_{i}-\bar{z_{i}}\right)}^{2},1\leq \gamma \leq N/\omega$$
- The quantum entropy of the coarse-grained vector is calculated and reconstructed, and the generalized multiscale quantum entropy formula is derived as shown in Equation (21).
  (21) $$E_{GMQE}(z,\omega ,m,\lambda )=E_{QE}(y_{g}^{\omega },m,\lambda )$$

Generalized multiscale quantum entropy (GMQE) largely retains the amplitude information of time series when examined across various scales. However, GMQE is significantly influenced by the coarse-grained time series. Under increasing scale factors, the GMQE curve exhibits substantial fluctuations, and this coarse-grained process will lead to the loss of fine-scale time information.

##### Refined Generalized Multiscale Quantum Entropy

During the coarse-graining process, the sequence length decays exponentially with the scale factor, resulting in unstable entropy values. To address this limitation, this paper proposes an improved refined generalized multiscale quantum entropy (RGMQE) composite technique.

The proposed technique constructs multiple coarse-grained representations through multivariate analysis, with ensemble averaging yielding the definitive output, thus obtaining a more accurate entropy value. The detailed calculation procedure of RGMQE is as follows:

- For the time series Z={z(ε),1≤ε≤N}, the generalized composite coarse-grained sequence $y_{g,k}^{\omega }=(y_{g,k,\gamma }^{\omega })$ of the original signal is calculated.
  (22) $$\begin{array}{l} y_{g,k,t}^{\omega }(\gamma )=\frac{1}{\omega }\sum_{\gamma \omega +k-1}^{i=(\gamma -1)\tau +k}{\left(z_{i}-\bar{z_{i}}\right)}^{2},1\leq \gamma \leq \frac{N}{\omega },1\leq k\leq \omega_{2}\leq \omega \\ \bar{z_{i}}=\frac{1}{\omega }\sum_{\omega -1}^{k=0}x_{i+k},\gamma ,k,\omega \in Z^{+} \end{array}$$
- The quantum entropy (QE) values of the generalized coarse-grained sequence $y_{g,k}^{\omega }$ are calculated.
- By calculating the mean of multiple QE values on the same scale, the RGMQE value under this scale τ is obtained, and the corresponding formula is as follows:
  (23) $$E_{RGMQE}(X,\omega ,m,\lambda )=\frac{1}{\tau }\sum E_{QE}(y_{g,k}^{\omega },m,\lambda )$$

**Figure 14:**
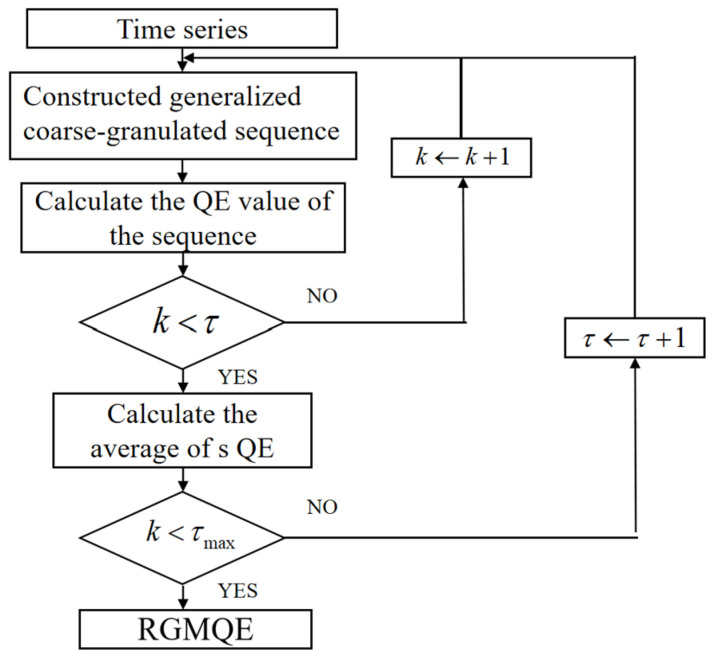
Flow diagram of RGMQE algorithm.

In RGMQE, the single coarse-grained time series used in GMQE is replaced with composite coarse-grained time series. By integrating the variance calculation approach with these composite series, the limitations of MQE and GMQE are effectively addressed. Based on Shannon’s information entropy theory, RGMQE values are constrained to the range of 0 to 1. The proposed technique quantifies multivariate dynamical complexity through scale-dependent entropy measures, evaluating system irregularity across multiple temporal resolutions. The detailed workflow of the proposed RGMQE algorithm is illustrated in Figure 14.

#### 3.2.2. Refined Generalized Multiscale Reverse Dispersion Entropy

##### Multiscale Reverse Dispersion Entropy

Reverse dispersion entropy (RDE) improves the stability of feature extraction by incorporating both the amplitude information from dispersion entropy and the distance information from inversely permuted entropy. On the other hand, multiscale reverse dispersion entropy (MRDE) provides a comprehensive description of time series complexity across multiple scales. The detailed computational steps are outlined below:

- Let time series z={z(ε),ε=1,2,…N}, using the given equation for defining a coarse-grained sequence $y^{\left(\omega \right)}=\{y^{\left(\omega \right)}(\gamma )\}$:
  (24) $$y^{\left(\omega \right)}(\gamma )=\frac{1}{\omega }\sum_{\varepsilon =(\gamma -1)\omega +1}^{\gamma r}z(\varepsilon ),\gamma =1,2,\ldots N/\omega$$
- Here, N represents the duration of z, while ω denotes the scaling factor, and the positive integer is taken.
- Inverse dispersion entropy is calculated, which coarse-grained sequences obtained under varying scale factors ω.
  (25) $$E_{MRDE}(z,c,m,\lambda ,\omega )=E_{RDE}(y^{\left(\omega \right)},c,m,\lambda )$$
  where $E_{MRDE}$ is multiscale inverse dispersion entropy; $E_{RDE}$ is the inverse dispersion of entropy. The parameters c,m,λ are the number of categories, embedding dimensions, and delay. When ω=1, MRDE is scale RDE.

##### Generalized Composite Multiscale Reverse Dispersion Entropy

MRDE constructs coarse-grained sequences by leveraging the mean characteristics of the data, capturing time series information across multiple scales. However, when extracting features from fault signals, mean processing tends to attenuate the kinetic mutation behavior of the original signal.

To address this limitation and better analyze dynamic changes in time series, this study introduces second-order moments as a replacement for the first-order moments employed in traditional coarse-grained methods. This approach leads to the development of generalized multiscale reverse dispersion entropy (GMRDE).

In step (1) of the MRDE calculation, for a given scale factor, to derive a new generalized series, only the variance of the original sequence is considered. Then, the RDE values for the generalized series across various scales are computed. The GMRDE values for the original series under these scale factors are determined.

GMRDE extends the coarse-graining process by replacing the mean value calculation with second-order moments. Theoretically, it surpasses the MRDE method but still exhibits certain limitations. During the multiscale process, data is divided into equal intervals, and variance is computed. While this approach is straightforward and efficient, the stability of entropy values is influenced by factors such as data length. Moreover, information from time series processed at alternative initial positions is overlooked.

To address these issues, a refined processing method [39] is employed to compute the probabilities of dispersion patterns for each coarse-grained sequence. These probabilities are then averaged, forming the basis for the calculation steps of refined generalized multiscale reverse dispersion entropy (RGMRDE), as outlined below:

- Consider the multivariate signal $[x=[x_{k,\varepsilon }](k=1,2,\ldots P);\varepsilon =1,2,\ldots ,N$, denoted as a matrix of P×N. Define the generalized coarse-grained sequence using Equation (26):
  (26) $$y_{k,l,\gamma }^{\left(\omega \right)}=\frac{1}{\tau }\sum_{\varepsilon =(\gamma -1)\omega +l}^{\gamma \omega +t-1}{\left(x_{k,\varepsilon }-\bar{x_{k,\varepsilon }}\right)}^{2}$$
  where P represents the number of signal channels; N signifies the duration of each channel within the multi-channel signal; $y_{k,l,\gamma }^{\left(\omega \right)}$ pertains to the given scale factor ω, specifically, considering the γ value associated with the l coarse-grained sequence within the k-channel data; $\bar{x_{k,\varepsilon }}$ is the average value of $x_{k,\varepsilon }$.
- For varying scale factors, the probabilities of all dispersion patterns in multi-channel generalized series are computed. These probabilities are determined by the ratio of embedding vectors mapped onto dispersion patterns, with consideration given to the overall count of elements within them.

Notably, while single-channel coarse-grained sequences are reconstructed into matrices, multi-channel sequences take the form of cells after reconstruction. To compute the dispersion mode probabilities, the cell must first be decomposed into a matrix before proceeding with further calculations.

- The mean value of the probability of a sequence ω of symbols is calculated, and the RGMRDE value is defined as follows based on Shannon’s entropy definition.
  (27) $$E_{RGMRDE}(z,c,m,\lambda ,\omega )=\frac{1}{\omega }\sum_{\gamma =2}^{\omega }E_{RDE}(y_{k,\gamma ,j}^{\left(\omega \right)},c,m,\lambda )$$
  (28) $$E_{RDE}(y_{k,\gamma ,j}^{\left(\omega \right)},c,m,\lambda )=\sum_{\gamma =1}^{c^{m}}{\left(\bar{P}(\pi_{v_{0}v_{1}\ldots v_{m-1}})-\frac{1}{C^{m}}\right)}^{2}=\sum_{\gamma =1}^{c^{m}}\bar{(P}(\pi_{v_{0}v_{1}\ldots v_{m-1}}){)}^{2}-\frac{1}{c^{m}}$$
  where $\bar{P}(\pi_{v_{0}v_{1}\ldots v_{m-1}})$ is the probability mean of the scattering pattern of the coarse-grained sequence π.

#### 3.2.3. Combinatorial Feature Extraction Based on Double-Layer Deep Extreme Learning Machine

**Figure 15:**
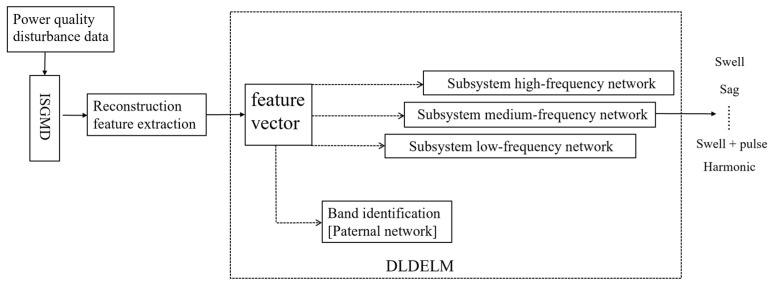
Identification structure diagram of DLDELM power quality disturbance.

Power quality disturbances exhibit distinct characteristic distributions, which can be categorized into amplitude changes and frequency changes. Specifically, Imf 1-Imf 3 primarily reflect frequency-related changes, while Imf 4 and Imf 5 represent amplitude-related changes. Extracting features solely using refined generalized multiscale quantum entropy (RGMQE) or refined generalized multiscale reverse dispersion entropy (RGMRDE) has inherent limitations. Therefore, feature extraction is performed based on the distribution and manifestation of different perturbations. RGMQE is more effective in capturing amplitude changes, whereas RGMRDE excels in describing frequency-related signals. To address this, a combined feature extraction method integrating multiscale quantum entropy and reverse dispersion entropy reconstruction is employed to extract features from Imfs associated with various disturbance types. Additionally, a double-layer deep extreme learning machine (DLDELM) model is utilized to identify the extracted low-dimensional feature values, as illustrated in Figure 15.

1. In the paternal DELM, IMF 2, IMF 3, IMF 4, and IMF 5 are extracted by RGMQE and RGMRDE, and the feature vectors shown in Formula (12) are used as inputs to identify the frequency band to which the output disturbance belongs.

(29) $$F_{\xi }={\left[F_{2},F_{3},H_{4},H_{5}\right]}^{T}$$

where ξ=1,2,3, and 1 is low-frequency disturbance, 2 is medium-frequency disturbance, and 3 is high-frequency disturbance.

2. Once the paternal DELM determines the disturbance’s frequency band, the corresponding sub-DELM identifies the specific disturbance type based on the result. The input layers of the low-, medium-, and high-frequency sub-DELMs contain 5, 6, and 8 neurons, respectively, while their output layers consist of 7, 7, and 12 neurons, respectively. Five-fold cross-validation was used to test the impact of the parent network and subnetwork structures on recognition accuracy, as shown in Figure 8, to verify the rationality of the selected parent network and subnetwork neuron numbers.

**Figure 16:**
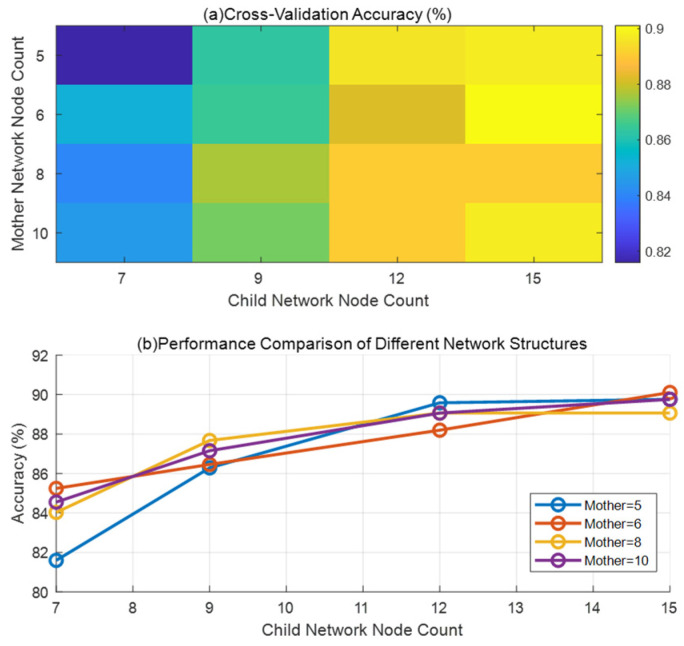
Double-layer deep limit learning machine (DLDELM) structure optimization experiment: (**a**) heat map (cross-validation accuracy); (**b**) comparison of parent network and child network structure performance.

As shown in Figure 16a,b, accuracy reaches its peak (ap proximately 0.95) when the number of parent network nodes is between 6 and 10 and the number of child network nodes is between 12 and 15, with this range exhibiting the best performance. As the number of child network nodes increases, accuracy gradually improves and stabilizes after reaching 12–15 nodes, with only limited further improvement beyond 15 nodes. The mother network typically performs the best when it has 8 or 10 nodes. However, having too few nodes (<5) or too many nodes (>20), as well as extreme parameter combinations (such as a mother network with 5 nodes paired with a child network with 7 nodes), can lead to a significant decrease in accuracy.

Based on the experimental results, we selected a network structure with a parent network consisting of 5/6/8 nodes: selecting 5 nodes may help to minimize computational complexity despite slightly lower performance; 6 nodes may be selected because the (6, 7) combination in Figure 16a performs well (approximately 0.88) despite not being optimal; and 8 nodes may be selected because Figure 16b shows this to be a performance-efficient configuration. The subnetwork consists of 7/7/12 nodes: the first two 7s correspond to the (6, 7) and (5, 7) combinations, respectively, where (6, 7) is a reasonable compromise, and (5, 7) is usable despite lower performance; 12 nodes were chosen because experiments showed that this is the point where performance begins to stabilize.

RGMQE extracted the characteristics of IMF 4 and IMF 5 from the low-frequency disturbed feature vector $F_{\varphi }$. The features of IMF 3, IMF 4, and IMF 5 were extracted by RGMQE and RGMRDE from the medium-frequency disturbed feature vector $F_{p}$. The features of Imf 1 to Imf 5 were extracted by RGMQE and RGMRDE from the high-frequency disturbed feature vector $F_{\delta }$. The input eigenvector expression of the subsystem network is shown as follows:

(30) $$\left\{\begin{array}{c} F_{\varphi }={\left[H_{4},H_{5}\right]}^{T}\\ F_{p}={\left[F_{3},H_{4},H_{5}\right]}^{T}\\ F_{\delta }={\left[F_{1},F_{2},F_{3},H_{4},H_{5}\right]}^{T} \end{array}\right.$$

The new dataset formed after feature reconstruction is $P^{\prime }=\{F_{\varphi },F_{p},F_{\delta },y\}$. The recognition model utilizes $P^{\prime }$ as its input data during both the training and evaluation phases and finally fuses into a composite power quality disturbance classification model so as to achieve the purpose of integrating the advantages of each feature classification and improving classification performance.

## 4. Simulation Analysis

### 4.1. Analysis of the Simulation Results of ISGMD

**Figure 17:**
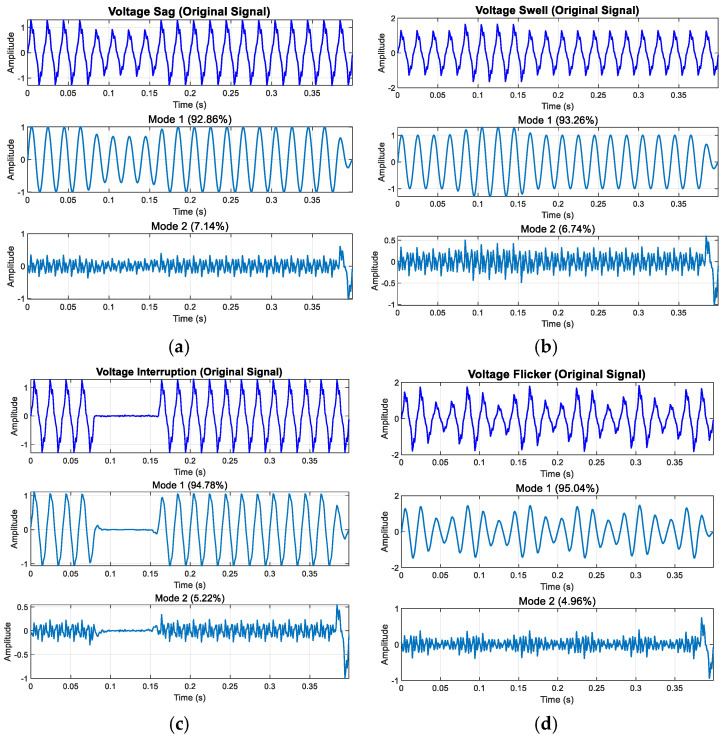
Modal decomposition results of low-frequency disturbances: (**a**) voltage sag; (**b**) voltage swell; (**c**) voltage interruption; (**d**) voltage flicker.

Figure 17 illustrates the decomposition results of low-frequency disturbances. As depicted, voltage swell, sag, interruption, and flicker exhibit amplitude characteristics in Component 1 and Component 2. Specifically, Figure 17c shows that during voltage interruption, the component amplitude within the perturbation interval approaches zero, reflecting the interruption’s distinctive feature.

Although the inherent marginal effect of modal decomposition introduces some distortion, the transient nature of interruption disturbances ensures minimal influence on the disturbance area. Consequently, this distortion has a negligible effect on the overall analysis and subsequent feature extraction.

**Figure 18:**
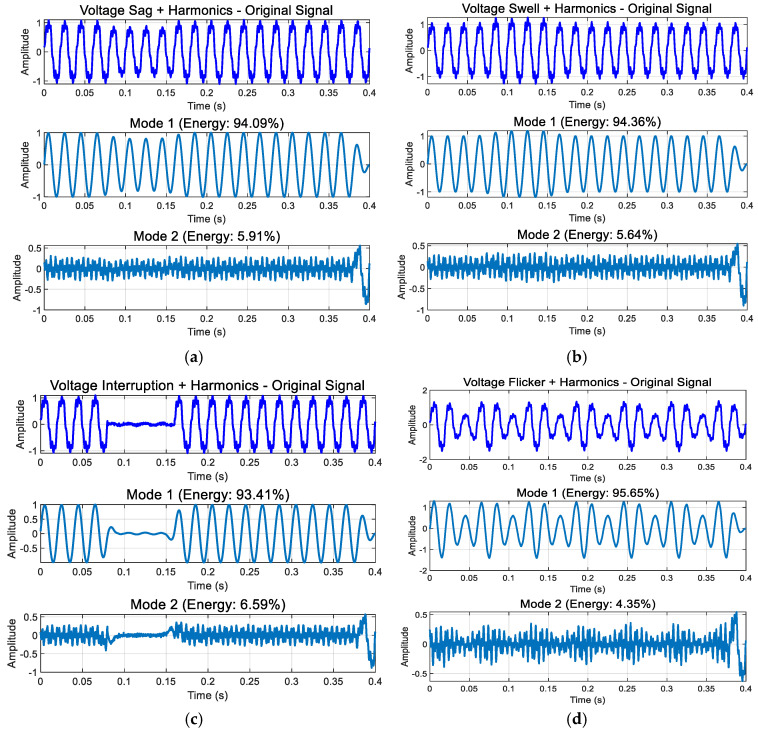
Modal decomposition results of IF disturbances: (**a**) sag + harmonic; (**b**) swell + harmonic; (**c**) interruption + harmonic; (**d**) flicker + harmonic.

Figure 18 presents the decomposition results of intermediate-frequency (IF) pertu-bations. As illustrated in Figure 18a, the presence of harmonics reveals distinct high-fr-quency harmonic components. Theoretically, high-frequency signal components are pr-marily embedded within the fundamental frequency signal. However, due to modal al-asing, a minor portion of the harmonic disturbance is decomposed into Component 2, while the key information is thoroughly decomposed.

**Figure 19:**
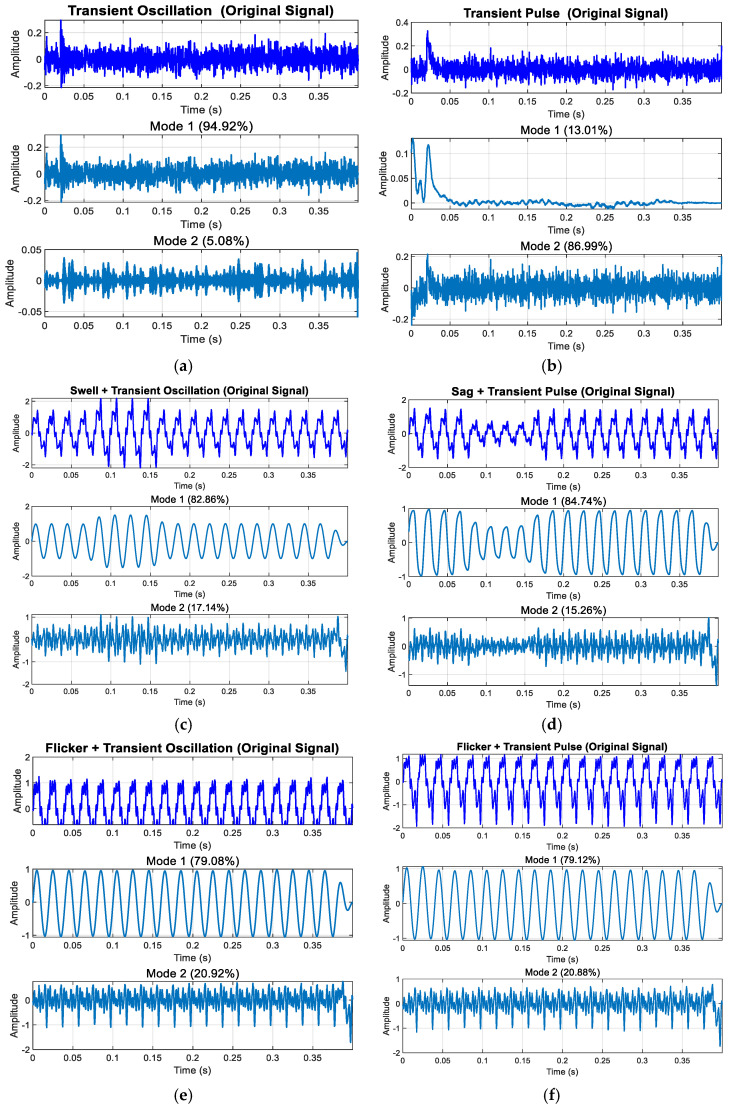
Modal decomposition results of high-frequency disturbances: (**a**) decomposition results of transient oscillation; (**b**) decomposition results of transient pulse; (**c**) decomposition results of swell + transient oscillation; (**d**) decomposition results of voltage sag + transient pulse; (**e**) decomp-sition results of flicker + transient oscillation; (**f**) decomposition results of voltage flicker + transient pulse.

Figure 19 displays the decomposition results of high-frequency disturbances. As shown in Figure 19a,b, voltage oscillations or pulses enhance mid- and high-frequency components, accompanied by amplitude variations during disturbances. Figure 19c,d demonstrate that during harmonic pulses or oscillations, the pulse, oscillation, harmonic, and fundamental wave are distinctly captured in Component 1, Component 2, and the original data.

From the decomposition results in Figure 17, Figure 18 and Figure 19, we can see the following: In the enhanced_SGMD function, the decomposition components are filtered by setting an energy proportion threshold of 0.5%, which is a key mechanism for ensuring the quality of signal decomposition. For voltage disturbance signals containing transient oscillation/pulse, SGMD typically decomposes the signal into multiple IMF components, including the highest-energy fundamental frequency component (50 Hz fundamental and its harmonics), the next highest-energy transient component (oscillation/pulse), and the extremely low-energy noise/residual components. Through energy threshold filtering, only the two significant components with energy exceeding the threshold are retained, while most low-energy IMF components are filtered out. It is important to note that all displayed energy percentages are normalized calculations relative to the retained significant components, not based on the total energy of the original signal. The filtered noise/residual components (e.g., 5%) do not participate in this percentage calculation, ensuring that the final retained components accurately reflect the signal’s primary characteristics.

### 4.2. Analysis of Identification Results

The outcomes derived from the improved symplectic geometric modal decomposition were subjected to feature extraction through the application of generalized composite multiscale quantum entropy and inverse dispersion entropy techniques, resulting in the formation of feature vectors. Following this, the data were classified and evaluated using an ensemble-based classification and recognition framework. Furthermore, a comparative assessment was conducted, focusing on factors such as model complexity, training duration, and accuracy.

#### 4.2.1. Comparison of Model Training

**Figure 20:**
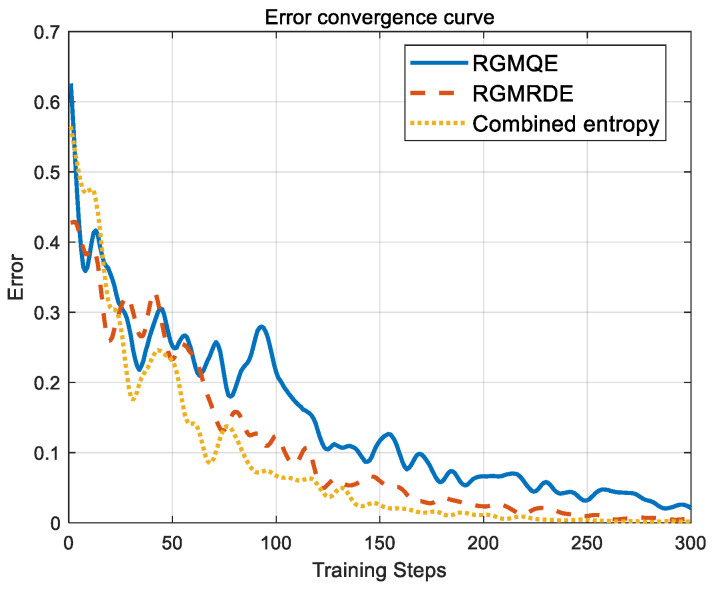
Output error convergence curve.

The integrated model is constructed, generating 110 training sets and 90 test sets. The feature vectors are trained using three distinct feature extraction methods. The convergence curves of the output errors for these three methods after training are illustrated in Figure 20. As depicted in this illustration, our proposed approach significantly enhances the training speed of the four subnetworks by 50 to 70 steps compared to feature extraction based on RGMQE and RGMRDE.

From the error convergence curve, it is evident that the proposed method demonstrates a notably faster training speed compared to both RGMQE and RGMRDE. In particular, the proposed method improves the training speed of the four subnetworks by 40 to 70 steps. This indicates that the proposed approach is more efficient in both feature extraction and model training, enabling it to achieve lower error values in fewer training iterations.

Three feature extraction methods were employed to conduct modeling training on the four algorithm models within the ensemble classification framework. Subsequently, comparisons were made based on the number of training steps, convergence time, number of network layers, and number of neurons in the models. Table 4 presents the complexity of the neural networks corresponding to the different extraction methods.

As illustrated in Table 4, in contrast to feature extraction utilizing multiscale quantum entropy and reverse dispersion entropy across various scales, the suggested approach reduces the average number of training steps by 70 and 40 steps, respectively. Additionally, the average convergence time is shortened by 16.4 s and 8.7 s, the number of hidden layers is decreased by one, and the number of neurons is reduced by 16. These results indicate that the features related to the feature extraction mechanism were optimized by the method we proposed, contributing to improved efficiency and reduced complexity in the modeling process.

#### 4.2.2. Comparative Analysis of Recognition Model Simulation Results

For 19 categories of power quality disturbance signals, 100 sets of test set signals were evaluated using three distinct feature extraction methods. Following this, the recognition accuracies of these methods were compared. The accuracy results for the three feature extraction methods are presented in Table 5.

As demonstrated in Table 5, compared to the methods of extracting feature vectors using composite multiscale quantum entropy and inverse dispersion entropy, the combined reconstruction feature extraction method achieves higher recognition accuracy for both single and compound perturbations.

To evaluate the performance of the three entropies, we performed confusion matrix visualization.

**Figure 21:**
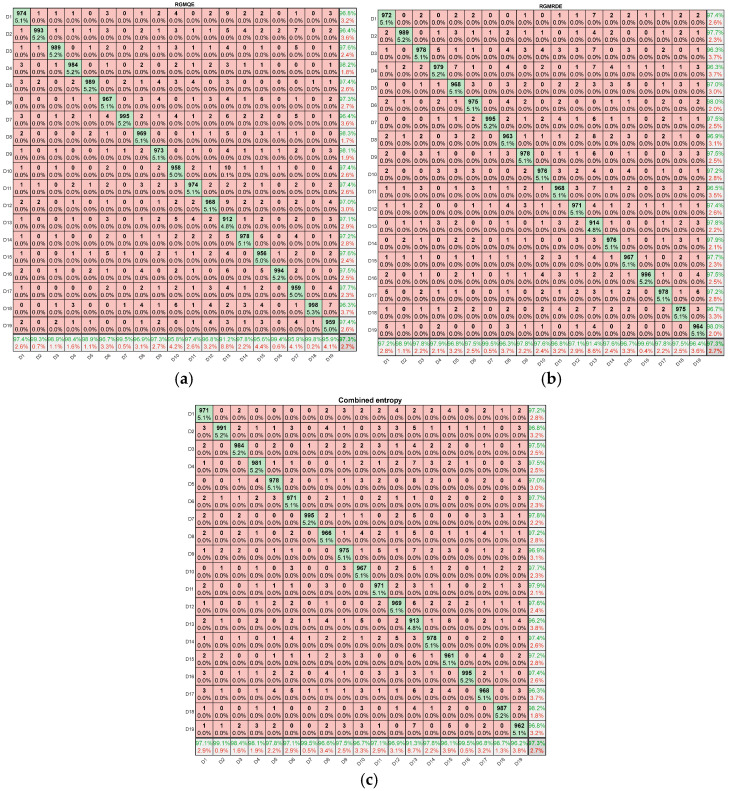
Confusion matrix of three types of entropy: (**a**) RGMQE; (**b**) RGMRDE; (**c**) combined entropy(Colors represent recall rates: Dark green indicates near-perfect classification (recall rate ≈ 100%); light green/yellow indicates moderate recall rates (50–75%); red indicates low recall rates (<50%), signifying severe classification errors.).

As shown in Figure 21, the RGMOE confusion matrix displays the preliminary classification results. The values along the diagonal are relatively dispersed, indicating lower classification accuracy for some categories; the RGMRDE confusion matrix shows significant improvement over the RGMQE matrix, with higher values concentrated along the diagonal, indicating enhanced classification performance. Figure 21c presents the classification results using the combined entropy method, which demonstrates overall superior performance. The values along the diagonal are generally higher, and misclassification in the non-diagonal regions is significantly reduced. Most categories achieve classification accuracy rates exceeding 95% under the combined entropy method, with several categories reaching over 97%.The combination entropy method, as the final optimization scheme, performs well in handling multi-category classification tasks and provides valuable reference for the design and optimization of classifiers.

#### 4.2.3. Ablation Experiment

To evaluate the effectiveness of each module, we conducted ablation experiments. The ablation experiments are divided into the following four models:

Model 1: SGMD + RGMQE/RGMRDE + DLDELM.
Model 2: ISGMD + RGMQE/RGMRDE + DLDELM.
Model 3: ISGMD + MQE/RDE + DLDELM.
Model 4: ISGMD + RGMQE/RGMRDE + ELM.

**Figure 22:**
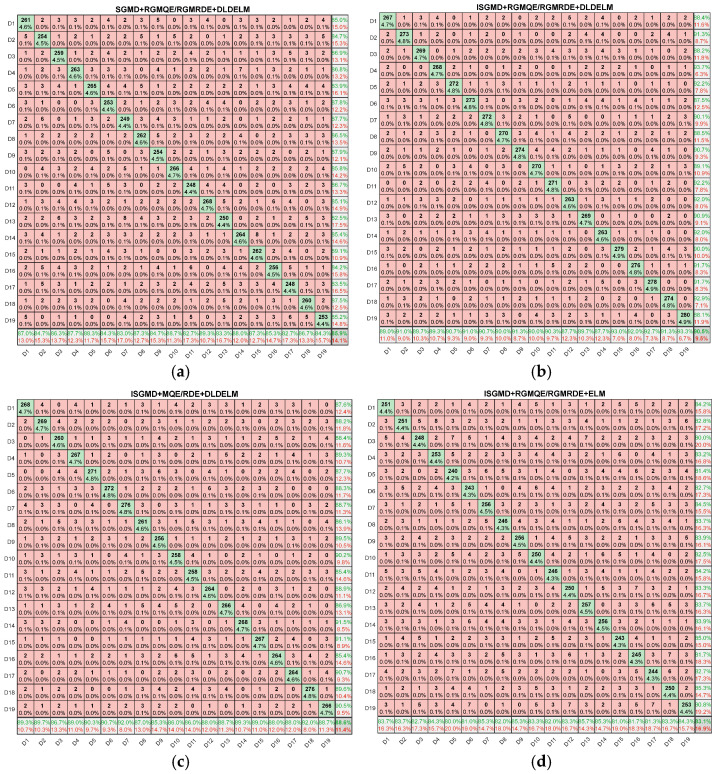
Confusion matrix: (**a**) SGMD + RGMQE/RGMRDE + DLDELM; (**b**) ISGMD + RGMQE/RGMRDE + DLDELM; (**c**) ISGMD + MQE/RDE + DLDELM; (**d**) ISGMD + RGMQE/RGMRDE + ELM. (Colors indicate the recall rate: dark green, near-perfect classification (recall ≈ 100%); light green/yellow, moderate recall (50–75%); red, low recall (<50%), indicating severe misclassification.)

In the ablation experiments, we used the 19 types of power quality disturbance signals listed in Table 2, with 100 samples per type, and analyzed the ablation experiment results using accuracy and confusion matrix analysis. The results are shown in Figure 22.

By comparing Model 2 and Model 4, the effectiveness of the DLDELM module can be demonstrated. In the ablation experiments, we used the 19 types of power quality disturbance signals listed in Table 2, with 100 samples per type, and analyzed the ablation experiment results using accuracy and confusion matrix analysis. A comparison of the effectiveness of each module is shown in Table 6 below.

As can be seen from Table 6, all improved modules (ISGMD, RGMQE/RGMRDE, DLDELM) have improved performance, with the DLDELM module showing the most significant improvement.

#### 4.2.4. Anti-Noise Simulation Analysis

For 19 types of electrical energy interference signals, 100 samples are taken from each group to generate 4 types of signals with 0, 20, 30, and 40 dB of Gaussian white noise added, respectively. Features were extracted using the method proposed in this study and subsequently recognized by a double-layer deep extreme learning machine (DLDELM). Table 7 displays the test results.

According to Table 7, the average accuracy exceeds 97%.

**Figure 23:**
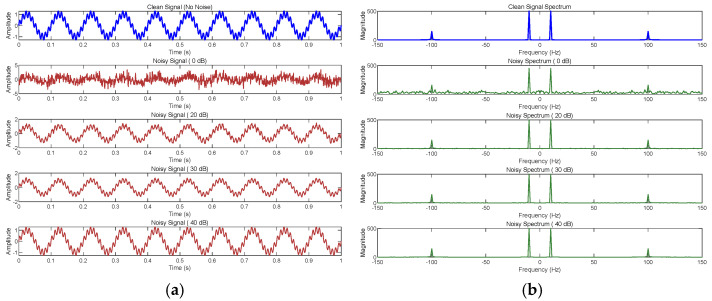
Noise visualization: (**a**) waveform diagram; (**b**) spectrum diagram.

Figure 23 visualizes the impact of noise by plotting the waveforms and frequency spectra of four signals. The frequency spectrum provides an intuitive, frequency-domain perspective on how noise affects signal quality.

As shown in Figure 23a, as noise increases from 0 dB to 40 dB, signal strength and clarity improve significantly. At 0 dB, the noise is comparable to the signal strength, and the sine wave is almost completely obscured by noise; at 20 dB, the signal begins to bcome visible but is still not clean enough; at 30 dB, the signal is stronger, and the waveform is clearer; and at 40 dB SNR, the signal is almost pure, with noise barely detectable. Figure 23b shows that at 0 dB, the noise is the strongest, and the peaks of the original signal are difficult to distinguish; at 20 dB, the noise begins to decrease, and the peaks gradually become visible; at 30 dB, the peaks are clear but still affected by noise; and at 40 dB, the signal spectrum is almost identical to the original clean signal, clearly distinguishable. Overall, this visualization result indicates that as noise decreases, signal components become increasingly easier to identify.

These results and analyses indicate that the combined reconstruction feature extraction method demonstrates high recognition accuracy and strong noise immunity across various noise environments.

## 5. Experimental Validation

**Figure 24:**
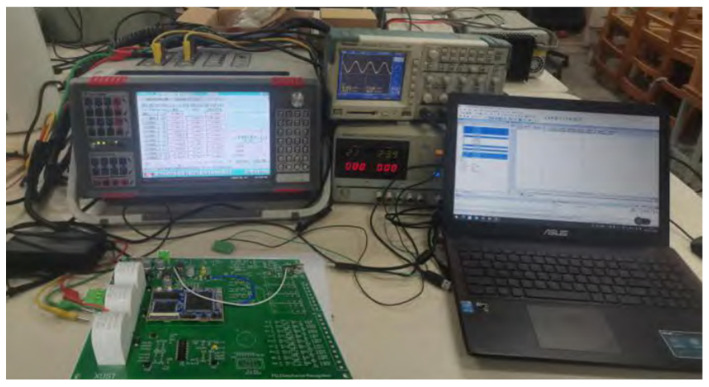
Experimental platform of power quality disturbance identification system.

In this section, an experimental platform for the power quality disturbance identification system is established, utilizing the STM32H743IIT6 as the core processor. To validate the platform, relevant operations are conducted. The setup is illustrated in Figure 24.

An ONLLY-AD461 relay protection test device serves as the disturbance signal generator. The disturbance signals captured by the oscilloscope are transmitted to the principal computer via the serial port, allowing for the real-time monitoring of the output signals. Additionally, the output normal voltage is set with an RMS value of 50 V, while the parameters of the remaining components are configured based on the simulation conditions.

### 5.1. Analysis of Model Decomposition Results

Due to hardware constraints, the laboratory bench’s signal source is unable to generate oscillation-related disturbances. Furthermore, multiple power quality disturbance (PQD) signals can only be simulated using the list function, which limits the simulation of multiple superimposed PQDs.

Building on prior simulation studies, this section applies the improved symplectic geometric mode decomposition to three types of triple perturbations and one type of quadruple perturbation. Figure 16 depicts the decomposition results.

**Figure 25:**
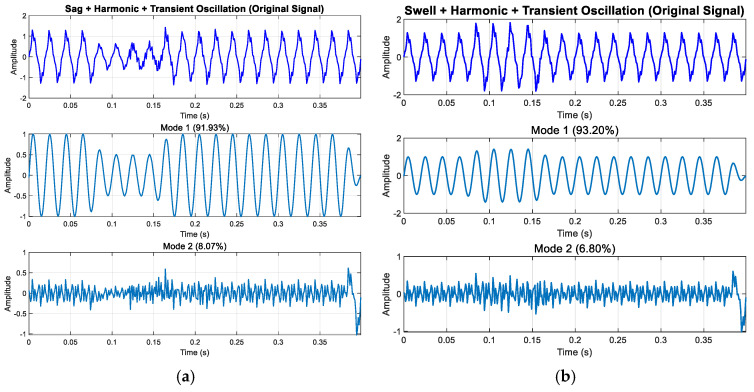

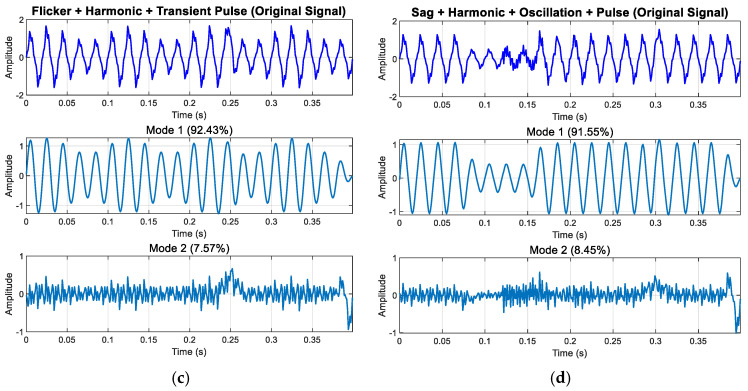
Experimental results of modal decomposition: (**a**) decomposition results of sag + ha-monic + transient oscillation; (**b**) decomposition results of swell + harmonic + transient oscillation; (**c**) decomposition results of flicker + harmonic + transient pulse; (**d**) decomposition results of sag + ha-monic + transient oscillation + transient pulse.

The signal decomposition analysis results in Figure 25 show that ISGMD can effectively separate complex power signals. Mode 1 accounts for over 90% of the total energy, primarily reflecting the fundamental and harmonic components of the signal, exhibiting regular sinusoidal characteristics; Mode 2 contains the remaining small energy components. Figure 25c,d further validate the effectiveness of this decomposition method, with Mode 1 occupying the majority of the signal’s total energy, successfully extracting the dominant periodic components of the signal.

This study aims to validate the efficacy of the proposed combined reconstruction and feature extraction method. A total of 60 sets of power quality disturbance signals were randomly generated per category of interference. The recognition accuracy of various di-turbance types is presented in Table 8.

As shown in Table 8, the combined reconstruction feature extraction method achieves a recognition accuracy of 98.1%, outperforming the other two feature extraction methods.

### 5.2. Comparison of Entropy Feature Performance

Based on the data in Table 8 of this manuscript, a performance comparison and statistical significance analysis of the three entropy feature methods were conducted under a 95% confidence interval, and a table listing the performance indicators of the 95% CI was compiled.

**Figure 26:**
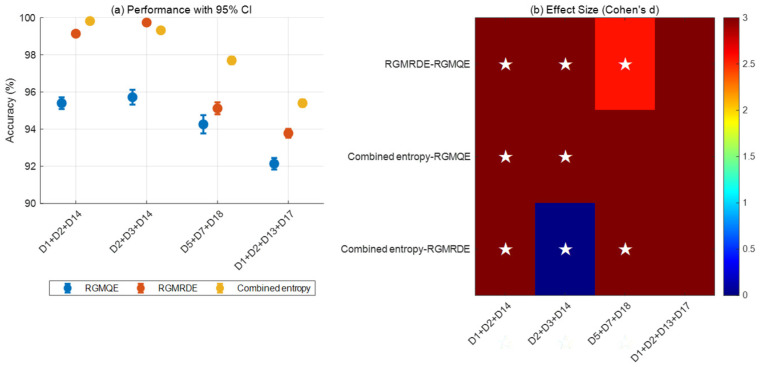
A performance comparison and effect size analysis of different methods in power quality disturbance detection. Note: An asterisk (★) indicates an effect size d > 1.5 (large effect). The color range 0–3 corresponds to Cohen’s d effect size standard (0.2: small effect; 0.5: medium effect; 0.8: large effect).

As shown in Figure 26a, there are significant differences in the performance of the three methods in power quality disturbance detection. Overall, the combined entropy method performs the best in most disturbance types, the RGMRDE method has intermediate performance, and the RGMOE method has relatively lower performance. Figure 26b further analyzes the significant differences between the methods, with the results showing the following: the red region indicates large effect sizes (d > 0.8), indicating significant differences between the methods, while the blue region indicates no significant differences between the methods. Analysis reveals that the combined entropy method demonstrates large positive effect sizes compared to the other two methods across multiple disturbance types, confirming its significant superiority over the RGMOE and RGMRDE methods; simultaneously, the RGMRDE and RGMOE methods also exhibit moderate effect size differences in certain disturbance types.

Table 9, Table 10, Table 11 and Table 12 show the average accuracy, standard deviation, 95% confidence interval, and coefficient of variation (CV) of the four signals under three entropy conditions.

Comprehensive analysis shows that the proposed combined entropy method has the best performance in power quality disturbance detection, and the performance differences between methods are statistically significant, especially when dealing with complex disturbances.

### 5.3. Hardware Platform Classification Results

Due to the limitations of the instrument signal source, this experiment was conducted using only 14 types of single and double composite disturbances for analysis. For each type of signal, 20 groups of signals were generated, with each sample having a sampling frequency of 12.8 kHz and a duration of 0.2 s. Table 13 details the algorithm’s experimental outcomes.

Since model formulation for PQD signals generated by the experimental console differs from the signal model created in the simulation experiment using the formula, the recognition rate of the algorithm decreases.

## 6. Conclusions

(1) In response to the non-stationary characteristics of power quality disturbance signals in power systems, this paper is based on the improved Sinusoidal Geometric Mode Decomposition (ISGMD) theory, which dynamically captures the time–frequency characteristics of signals through an adaptive decomposition mechanism. Unlike traditional decomposition methods with predefined frequency bands, this method can adaptively decompose signals into several IMF components, effectively extracting the intrinsic modal characteristics of non-stationary signals. Through this adaptive decomposition strategy, the effectiveness of each component is significantly improved, and the negative impact of feature vector redundancy on recognition performance is effectively reduced.
(2) In terms of feature extraction, this paper proposes a combined reconstruction feature extraction method that integrates RGMQE and RGMRDE by qualitatively analyzing the disturbance feature distribution and representation differences. This method constructs high-precision, low-dimensional feature vectors and uses the DLDELM model for simulation recognition. Experimental verification shows that the constructed feature extraction framework and recognition model have low complexity, high recognition accuracy, and significant noise resistance.
(3) Under the comprehensive classification and combination reconstruction model, this method achieved an overall identification rate of 97.43% for power quality disturbances in different noise intensity environments and an even more outstanding performance of 99.2% in a noise-free environment, demonstrating excellent noise robustness. In particular, the accurate identification results of 14 types of power quality disturbance signals generated based on the hardware platform further verified the powerful generalization ability of this model. Compared with existing CNN + LSTM methods, the recognition accuracy was improved by 3.7%, and its performance in small sample scenarios is also significantly better than traditional CNN and LSTM single models. The simulation database (containing 63 types of power quality disturbances) achieved an average classification accuracy rate of 99.10% in a 30 dB white noise environment, and the average accuracy rate of the hardware platform test data also reached 99.03%, fully proving the reliability and effectiveness of the proposed method in actual engineering applications.

## Figures and Tables

**Table 1:** Performance indicators of SGMD and ISGMD.

Algorithm	Noise Amplitude	Number of Noises	Decomposition Time	Number of Components	IOO	ICC
SGMD	0.2398	2	0.0182	6	1.3902	0.9994
ISGMD	0.2398	2	0.0385	6	1.4006	0.9996

**Table 2:** Basic signal model for power quality disturbances.

Disturbance	Type Number	Signal Model Parameter Description
harmonic	D1	$\begin{array}{l} V(t)=\sin(wt)+a_{3}\sin(3wt+\phi_{3})\\ +a_{5}\sin(5wt+\phi_{5})+a_{7}\sin(7wt+\phi_{7}) \end{array}$ $\begin{array}{l} \alpha_{n}=0\sim 0.15(n=3,5,7)\\ \varphi_{n}=0\sim 2\pi (n=3,5,7) \end{array}$
voltage sag	D2	$V(t)=(1-\alpha (u(t-t_{1})-u(t-t_{2})))\sin(\omega t)$ $\begin{array}{l} \alpha =0.1\sim 0.9;\\ t_{2}-t_{1}=4T\sim 9T \end{array}$
voltage swell	D3	$V(t)=(1+\alpha (u(t-t_{1})-u(t-t_{2})))\sin(\omega t)$ $\begin{array}{l} \alpha =0.1\sim 0.9;\\ t_{2}-t_{1}=4T\sim 9T \end{array}$
voltage interrupt	D4	$V(t)=(1-\alpha (u(t-t_{1})-u(t-t_{2})))\sin(\omega t)$ $\begin{array}{l} \alpha =0.9\sim 1;\\ t_{2}-t_{1}=4T\sim 9T \end{array}$
voltage flicker	D5	$V(t)=(1+\alpha_{f}\sin(\beta \omega t))\sin(\omega t)$ $\begin{array}{l} \alpha_{f}=0.3\sim 0.5;\\ \beta =0.1\sim 0.4 \end{array}$
transient oscillation	D6	$\begin{array}{l} V(t)=\sin(\omega t)+\alpha_{2}e^{-\frac{t-t_{3}}{\tau }}\sin\{\omega_{n}(t-t_{3})\}\\ {}*\{u(t-t_{3})-u(t-t_{4})\} \end{array}$ $\begin{array}{l} \alpha_{2}=0.1\sim 0.8,\tau =0.008\sim 0.04;\\ t_{4}-t_{3}=0.05T\sim 3T;f_{n}=300\sim 900\;\mathrm{Hz} \end{array}$
transient pulse	D7	$\begin{array}{l} V(t)=\sin(\omega t)+\alpha_{2}e^{-\frac{t-t_{3}}{\tau }}\\ {}*\{u(t-t_{3})-u(t-t_{4})\} \end{array}$ $\begin{array}{l} \alpha_{2}=0.1\sim 0.8,\tau =0.008\sim 0.04;\\ t_{4}-t_{3}=0.05T\sim 3T \end{array}$
harmonic + sag	D8	$\begin{array}{l} V(t)=(1-\alpha (u(t-t_{1})-u(t-t_{2})))\sin(\omega t)\\ +\alpha_{3}\sin(3\omega t+\phi_{3})+\alpha_{5}\sin(5\omega t+\phi_{5})\\ +\alpha_{7}\sin(7\omega t+\phi_{7}) \end{array}$ $\begin{array}{l} \alpha =0.1\sim 0.9,t_{2}-t_{1}=4T\sim 9T,\alpha_{3}=0\sim 0.15\\ \alpha_{5}=0\sim 0.15,\alpha_{7}=0\sim 0.15,\\ \phi_{3}=0\sim 2\pi ,\phi_{5}=0\sim 2\pi ,\phi_{7}=0\sim 2\pi \end{array}$
harmonic + swell	D9	$\begin{array}{l} V(t)=(1+\alpha (u(t-t_{1})-u(t-t_{2})))\sin(\omega t)\\ +\alpha_{3}\sin(3\omega t+\phi_{3})+\alpha_{5}\sin(5\omega t+\phi_{5})\\ +\alpha_{7}\sin(7\omega t+\phi_{7}) \end{array}$ $\begin{array}{l} \alpha =0.1\sim 0.9,t_{2}-t_{1}=4T\sim 9T,\alpha_{3}=0\sim 0.15\\ \alpha_{5}=0\sim 0.15,\alpha_{7}=0\sim 0.15,\\ \phi_{3}=0\sim 2\pi ,\phi_{5}=0\sim 2\pi ,\phi_{7}=0\sim 2\pi \end{array}$
harmonic + interrupt	D10	$\begin{array}{l} V(t)=(1-\alpha (u(t-t_{1})-u(t-t_{2})))\sin(\omega t)\\ +\alpha_{3}\sin(3\omega t+\phi_{3})+\alpha_{5}\sin(5\omega t+\phi_{5})\\ +\alpha_{7}\sin(7\omega t+\phi_{7}) \end{array}$ $\begin{array}{l} \alpha =0.9\sim 1,t_{2}-t_{1}=4T\sim 9T,\alpha_{3}=0\sim 0.15\\ \alpha_{5}=0\sim 0.15,\alpha_{7}=0\sim 0.15,\\ \phi_{3}=0\sim 2\pi ,\phi_{5}=0\sim 2\pi ,\phi_{7}=0\sim 2\pi \end{array}$
harmonic + flicker	D11	$\begin{array}{l} V(t)=(1+\alpha_{f}\sin(\beta \omega t)\sin(\omega t)\\ +\alpha_{3}\sin(3\omega t+\phi_{3})+\alpha_{5}\sin(5\omega t+\phi_{5})\\ +\alpha_{7}\sin(7\omega t+\phi_{7}) \end{array}$ $\begin{array}{l} \alpha_{f}=0.3\sim 0.5,\beta =0.1\sim 0.4,\alpha_{3}=0\sim 0.15\\ \alpha_{5}=0\sim 0.15,\alpha_{7}=0\sim 0.15,\\ \phi_{3}=0\sim 2\pi ,\phi_{5}=0\sim 2\pi ,\phi_{7}=0\sim 2\pi \end{array}$
sag + oscillation	D12	$\begin{array}{l} V(t)=(1-\alpha (u(t-t_{1})-u(t-t_{2})))\sin(\omega t)\\ +\alpha_{2}e^{-\frac{t-t_{3}}{\tau }}\sin\{(\omega_{n}(t-t_{3})\}\{u(t-t_{3})-u(t-t_{4})\} \end{array}$ $\begin{array}{l} \alpha =0.1\sim 0.9,t_{2}-t_{1}=4T\sim 9T,\alpha_{2}=0.1\sim 0.8\\ \tau =0.008\sim 0.04,\\ t_{4}-t_{3}=0.05T\sim 3T,f_{n}=300\sim 900\;\mathrm{Hz} \end{array}$
swell + oscillation	D13	$\begin{array}{l} V(t)=(1+\alpha (u(t-t_{1})-u(t-t_{2})))\sin(\omega t)\\ +\alpha_{2}e^{-\frac{t-t_{3}}{\tau }}\sin\{(\omega_{n}(t-t_{3})\}\{u(t-t_{3})-u(t-t_{4})\} \end{array}$ $\begin{array}{l} \alpha =0.1\sim 0.9,t_{2}-t_{1}=4T\sim 9T,\alpha_{2}=0.1\sim 0.8\\ \tau =0.008\sim 0.04,\\ t_{4}-t_{3}=0.05T\sim 3T,f_{n}=300\sim 900\;\mathrm{Hz} \end{array}$
flicker + oscillation	D14	$\begin{array}{l} V(t)=(1+\alpha_{f}\sin(\beta \omega t)\sin(\omega t)\\ +\alpha_{2}e^{-\frac{\left(t-t_{3}\right)}{\tau }}\sin\{\omega_{n}(t-t_{3})\}\{u(t-t_{3})-u(t-t_{4})\} \end{array}$ $\begin{array}{l} \alpha_{f}=0.3\sim 0.5,\beta =0.1\sim 0.4,\\ \alpha_{2}=0.1\sim 0.8,\tau =0.008\sim 0.04,\\ t_{4}-t_{3}=0.05T\sim 3T,f_{n}=300\sim 900\;\mathrm{Hz} \end{array}$
harmonic + oscillation	D15	$\begin{array}{l} V(t)=\sin(\omega t)+\alpha_{3}\sin(3\omega t+\phi_{3})+\\ \alpha_{5}\sin(5\omega t+\phi_{5})+\alpha_{7}\sin(7\omega t+\phi_{7})+\\ \alpha_{2}e^{-\frac{\left(t-t_{3}\right)}{\tau }}\sin\{\omega_{n}(t-t_{3})\}\cdot \{u(t-t_{3})-u(t-t_{4}\} \end{array}$ $\begin{array}{l} \alpha_{3}=0\sim 0.15,\alpha_{5}=0\sim 0.15,\alpha_{7}=0\sim 0.15,\\ \phi_{3}=0\sim 2\pi ,\phi_{5}=0\sim 2\pi ,\phi_{7}=0\sim 2\pi ,f_{n}=300\sim 900\;\mathrm{Hz}\\ \alpha_{2}=0.1\sim 0.8,\tau =0.008\sim 0.04,t_{4}-t_{3}=0.05T\sim 3T \end{array}$
sag + pulse	D16	$\begin{array}{l} V(t)=(1-\alpha (u(t-t_{1})-u(t-t_{2})))\sin(\omega t)\\ +\alpha_{2}e^{-\frac{t-t_{3}}{\tau }}\{u(t-t_{3})-u(t-t_{4})\} \end{array}$ $\begin{array}{l} \alpha =0.1\sim 0.9,t_{2}-t_{1}=4T\sim 9T,\alpha_{2}=1\sim 10\\ \tau =0.008\sim 0.04,t_{4}-t_{3}=0.05T\sim 3T \end{array}$
swell + pulse	D17	$\begin{array}{l} V(t)=(1+\alpha (u(t-t_{1})-u(t-t_{2})))\sin(\omega t)\\ +\alpha_{2}e^{-\frac{t-t_{3}}{\tau }}\{u(t-t_{3})-u(t-t_{4})\} \end{array}$ $\begin{array}{l} \alpha =0.1\sim 0.9,t_{2}-t_{1}=4T\sim 9T,\alpha_{2}=1\sim 10\\ \tau =0.008\sim 0.04,t_{4}-t_{3}=0.05T\sim 3T \end{array}$
flicker + pulse	D18	$\begin{array}{l} V(t)=(1+\alpha_{f}\sin(\beta \omega t)\sin(\omega t)\\ +\alpha_{2}e^{-\frac{\left(t-t_{3}\right)}{\tau }}\{u(t-t_{3})-u(t-t_{4})\} \end{array}$ $\begin{array}{l} \alpha_{f}=0.3\sim 0.5,\beta =0.1\sim 0.4,\alpha_{2}=1\sim 10,\\ \tau =0.008\sim 0.04,t_{4}-t_{3}=0.05T\sim 3T \end{array}$
harmonic + pulse	D19	$\begin{array}{l} V(t)=\sin(\omega t)+\alpha_{3}\sin(3\omega t+\phi_{3})+\\ \alpha_{5}\sin(5\omega t+\phi_{5})+\alpha_{7}\sin(7\omega t+\phi_{7})\\ +\alpha_{2}e^{-\frac{\left(t-t_{3}\right)}{\tau }}\{u(t-t_{3})-u(t-t_{4})\} \end{array}$ $\begin{array}{l} \alpha_{3}=0\sim 0.15,\alpha_{5}=0\sim 0.15,\alpha_{7}=0\sim 0.15,\\ \phi_{3}=0\sim 2\pi ,\phi_{5}=0\sim 2\pi ,\phi_{7}=0\sim 2\pi ,\\ \alpha_{2}=1\sim 10,\tau =0.008\sim 0.04,t_{4}-t_{3}=0.05T\sim 3T \end{array}$

**Table 3:** Pearson correlation coefficients of IMFs.

Disturbance	IMF 1	IMF 2	IMF 3	IMF 4	IMF 5	IMF 6
D1	0.09	0.15	**0.35**	**0.95**	**0.94**	0.03
D2	0	0.02	0.04	**0.98**	**0.97**	0.06
D3	0.02	0.03	0.07	**0.97**	**0.94**	0
D4	0.01	0.02	0.09	**0.96**	**0.91**	0.03
D5	0.03	0.05	0.06	**0.97**	**0.98**	0.07
D6	**0.35**	**0.36**	**0.41**	**0.92**	**0.94**	0.08
D7	**0.33**	**0.37**	**0.55**	**0.90**	**0.92**	0.04
D8	0.02	0.03	**0.34**	**0.97**	**0.94**	0.05
D9	0.01	0.06	**0.40**	**0.90**	**0.85**	0.04
D10	0.09	0.11	**0.45**	**0.95**	**0.95**	0.04
D11	0.05	0.08	**0.43**	**0.96**	**0.97**	0.05
D12	**0.31**	**0.35**	**0.60**	**0.95**	**0.93**	0.06
D13	**0.31**	**0.36**	**0.65**	**0.96**	**0.95**	0.01
D14	**0.28**	**0.38**	**0.64**	**0.97**	**0.96**	0.09
D15	**0.40**	**0.60**	**0.65**	**0.96**	**0.95**	0.01
D16	**0.45**	**0.65**	**0.55**	**0.94**	**0.95**	0.08
D17	**0.35**	**0.45**	**0.70**	**0.86**	**0.88**	0.06
D18	**0.33**	**0.42**	**0.51**	**0.98**	**0.99**	0.02
D19	**0.38**	**0.55**	**0.56**	**0.99**	**0.95**	0.05

**Table 4:** Comparison of model complexity of different feature extraction methods.

Feature Extraction Methods	Step Count	Convergence Time/s	Number of Layers	Number of Neurons
RGMQE	150	35.2	4	64
RGMRDE	120	28.5	3	48
Combined entropy	80	19.8	3	48

**Table 5:** Comparative recognition accuracy across feature extraction methods.

Disturbance	RGMQE	RGMRDE	Combined Entropy
D1	97.4	97.2	97.1
D2	99.3	98.9	99.1
D3	98.9	97.8	98.4
D4	98.4	97.9	98.1
D5	98.9	96.8	97.8
D6	96.7	97.5	97.1
D7	99.5	99.5	99.5
D8	96.9	96.3	96.6
D9	97.3	97.8	97.5
D10	95.8	97.6	96.7
D11	97.4	96.8	97.1
D12	96.8	97.1	96.9
D13	91.2	91.4	91.3
D14	97.8	97, 6	97.8
D15	95.6	96.7	96.1
D16	99.4	99.6	99.5
D17	95.9	97.8	96.8
D18	99.8	97.5	98.7
D19	95.9	96.4	96.2

**Table 6:** Comparison of module effectiveness analysis.

Module	Comparison Model	Accuracy	Improvement
ISGMD	Model 1 (SGMD)	84.91%	-
Module	Model 2 (ISGMD)	90.00%	+5.09%
			
RGMQE/RGMRDE	Model3 (MQE/RDE)	89.89%	-
Module	Model2 (RGMQE/RGMRDE)	90.00%	+2.11%
			
DLDELM	Model 4 (ELM)	81.93%	-
Module	Model 2 (DIDELM)	90.00%	+8.07%

**Table 7:** Accuracy of noise environment recognition.

Disturbance	0 dB	20 dB	30 dB	40 dB
D1	99.2	98.5	98.9	98.7
D2	98.9	96.7	97.8	97.5
D3	98.7	96.2	97.5	97.3
D4	96.8	97.5	96.5	96.5
D5	97.9	97.8	98.2	98.1
D6	90.9	88.4	89.6	88.9
D7	98.8	89.8	98.6	97.9
D8	98.2	94.2	98.5	98.2
D9	98.5	96.8	98.3	98.1
D10	98.8	96.1	98.2	97.8
D11	98.2	97.5	97.9	97.7
D12	98.9	95.6	97.3	98.2
D13	98.1	96.9	97.8	97.5
D14	97.7	94.8	97.4	96.9
D15	96.7	95.6	96.7	96.7
D16	97.1	95.3	96.9	96.5
D17	98.4	96.7	98.3	98.1
D18	99.3	97.8	99.1	98.9
D19	98.9	96.8	97.7	94.6
AVERAGE ACCURACY (%)	97.89	97.73	97.43	97.05

**Table 8:** Accuracy of power quality disturbance identification on hardware platform.

Disturbance	RGMQE	RGMRDE	Combined Entropy
D1 + D2 + D14	95.432	99.176	99.875
D2 + D3 + D14	95.828	99.765	99.351
D5 + D7 + D18	94.158	95.067	97.752
D1 + D2 + D13 + D17	92.172	93.831	95.471
Average accuracy	94.398	96.959	98.112

**Table 9:** D1 + D2 + D14.

Feature Extraction Methods	Mean	Std	CI_95%	CV
RGMQE	95.4	0.255	[95.08, 95.72]	0.3
RGMRDE	99.14	0.114	[99.00, 99.28]	0.1
Combined entropy	99.82	0.084	[99.72, 99.92]	0.1

**Table 10:** D2 + D3 + D14.

Feature Extraction Methods	Mean	Std	CI_95%	CV
RGMQE	95.72	0.319	[95.32, 96.12]	0.3
RGMRDE	99.74	0.089	[99.63, 99.85]	0.1
Combined entropy	99.32	0.084	[99.22, 99.42]	0.1

**Table 11:** D5 + D7 + D18.

Feature Extraction Methods	Mean	Std	CI_95%	CV
RGMQE	94.26	0.397	[93.77, 94.75]	0.4
RGMRDE	95.12	0.259	[94.80, 95.44]	0.3
Combined entropy	97.7	0.158	[97.50, 97.90]	0.2

**Table 12:** D1 + D2 + D13 + D17.

Feature Extraction Methods	Mean	Std	CI_95%	CV
RGMQE	92.14	0.251	[91.83, 92.45]	0.3
RGMRDE	93.78	0.192	[93.54, 94.02]	0.2
Combined entropy	95.4	0.158	[95.20, 95.60]	0.2

**Table 13:** Detection results of perturbation experiments.

Disturbance	Classification Accuracy	Disturbance	Classification Accuracy
voltage sag	98.1%	pulse + flicker	97.2%
voltage swell	97.7%	flicker + oscillation	97.1%
oscillation	96.8%	interrupt + harmonic	98.4%
voltage pulse	96.3%	harmonic + flicker	98.1%
harmonic	99.5%	harmonic + oscillation	97.5%
interrupt	96.4%	voltage swell + pulse	97.9%
voltage flicker	95.8%	voltage sag + flicker	96.7%

## Data Availability

The data from this study can be obtained upon request to the corresponding author.

## Abbreviations

PQD	Power Quality Disturbance
GAFs	Gramian Angular Fields
GA	Genetic Algorithm
GAN	Generative Adversarial Network
DBNs	Deep Belief Networks
BiLSTM	Bi-directional Long Short-Trem Memory
ISGMD	Improved Symplectic Geometric Mode Decomposition
ISGMCs	Initial Symplectic Geometric Mode Components
RGMQE	Refined Generalized Multiscale Quantum Entropy
PCC	Pearson Correlation Coefficient
GMRDE	Generalized Multiscale Reverse Dispersion Entropy
IMF	Intrinsic Mode Function
RGMRDE	Refined Generalized Multiscale Reverse Dispersion Entropy
DLDELM	Double-layer Deep Extreme Learning Machine
RMS	Root Mean Square

## References

[1] Yu D., Han X., Wang W., Zhang H., Xiong P., Dai K. (2025). Power Supply for the Projectile Borne Electromechanical System: A Review. Green Energy Intell. Transp..

[2] Samanta I.S., Mohanty S., Parida S.M., Rout P.K., Panda S., Bajaj M., Blazek V., Prokop L., Misak S. (2025). Artificial Intelligence and Machine Learning Techniques for Power Quality Event Classification: A Focused Review and Future Insights. Results Eng..

[3] Cui L., Wang Z., Liu Y., Cao G. (2024). How Does Data-Driven Supply Chain Analytics Capability Enhance Supply Chain Agility in the Digital Era. Int. J. Prod. Econ..

[4] Li H., Zhang C. (2025). Hydrogen Saving and Power Improvement for Proton Exchange Membrane Fuel Cells via Optimum Stack Operating Temperature Tracking with Enhanced Performance. Int. J. Hydrogen Energy.

[5] Mohanty A., Ray P.K., Das S.R., Soudagar M.E., Ramesh S., Khan T.M.Y., Ali M.M., Bashir M.N. (2024). Enhancing Power Quality in Contemporary Utility Systems: A Comprehensive Analysis of Active Power Filters and Control Strategies. Energy Rep..

[6] Abdullah H.A., Hanif M.U., Hassan M.U., Shahid J.M., Khan S.A., Ali A. (2025). Improved Damage Assessment of Bridges Using Advanced Signal Processing Techniques of CEEMDAN-EWT and Kernel PCA. Eng. Struct..

[7] Ho S., Jang G., Kwon S. (2010). Time-Frequency Analysis of Power-Quality Disturbances via the Gabor-Wigner Transform. IEEE Trans. Power Deliv..

[8] Fischer J.R., González S.A., Carugati I., Judewicz M.G., Carrica D.O. (2015). Control Directo de Potencia Predictivo Robusto con Sincronismo Intrínseco. Rev. Iberoam. Autom. Inform. Ind..

[9] Santoso S., Powers E.J., Grady W.M., Hofmann P. (2002). Power quality assessment via wavelet transform analysis. IEEE Trans. Power Deliv..

[10] Huang N.E., Shen Z., Long S.R., Wu M.C., Shih H.H., Zheng Q., Yen N.-C., Tung C.C., Liu H.H. (1998). The empirical mode decomposition and the Hilbert spectrum for nonlinear and non-stationary time series analysis. Proc. R. Soc. A.

[11] Ge J., Wang L., Gui K., Ye L. (2023). Temperature Interpretation Method for Temperature Indicating Paint Based on Spectrogram. Measurement.

[12] Cao F., Gao F., Yuan D., Liu J. (2024). Multistep Asymptotic Pre-Training Strategy Based on PINNs for Solving Steep Boundary Singular Perturbation Problems. Comput. Methods Appl. Mech. Eng..

[13] Sun Z., Li S., Yang H. (2024). Precise Disturbance Localization of Long-Distance Fiber Interferometer Vibration Sensor Based on an Improved Time Frequency Variation Feature Extraction Scheme. Infrared Phys. Technol..

[14] Nazari A., Jamshidi M., Roozbahani A., Golparvar B. (2025). Groundwater Level Forecasting Using Empirical Mode Decomposition and Wavelet-Based Long Short-Term Memory Neural Networks. Groundw. Sustain. Dev..

[15] Ni C., Chen H., Chen Y., Yao Y., Li L. (2023). Power Quality Disturbances Identification Based on Adaptive Symplectic Geometric Mode Decomposition and Improved Marine Predators Algorithm. Electr. Power Syst. Res..

[16] Salles R.S., Ribeiro P.F. (2023). The Use of Deep Learning and 2-D Wavelet Scalograms for Power Quality Disturbances Classification. Electr. Power Syst. Res..

[17] Wang L., Zhou Y., Sun X., Wu S., Xia L., Sun J., Zha Y., Yang P. (2024). Retrieval of Chromium and Mercury Concentrations in Agricultural Soils: Using Spectral Information, Environmental Covariates, or a Fusion of Both. Ecol. Indic..

[18] Wang Y., Huang Q., Xie Z., Wang M., Bao W. (2023). Evaluation on Game Concentration with Multi-Scale Fuzzy Entropy Based on EEG Signals. Entertain. Comput..

[19] Caicedo J.E., Agudelo-Martínez D., Rivas-Trujillo E., Meyer J. (2023). A Systematic Review of Real-Time Detection and Classification of Power Quality Disturbances. Prot. Control Mod. Power Syst..

[20] Bilgundi S.K., Sachin R., Pradeepa H., Nagesh H.B., Kumar M.L. (2022). Grid Power Quality Enhancement Using an ANFIS Optimized PI Controller for DG. Prot. Control Mod. Power Syst..

[21] Gülmez B. (2025). GA-Attention-Fuzzy-Stock-Net: An Optimized Neuro-Fuzzy System for Stock Market Price Prediction with Genetic Algorithm and Attention Mechanism. Heliyon.

[22] Ning F., Cheng Z., Meng D., Wei J. (2021). A Framework Combining Acoustic Features Extraction Method and Random Forest Algorithm for Gas Pipeline Leak Detection and Classification. Appl. Acoust..

[23] Hu X., Liu K., Jin X., Zhang D., Chen C., Xu T., Jiang J., Liu T. (2024). Wide-Frequency-Range Vibration Positioning Based on Adaptive TQWT for Long-Distance Asymmetric Interferometer Sensor. Opt. Laser Eng..

[24] Ruan Z., Xiao X., Hu W. (2022). Multiple Power Quality Disturbance Classification Feature Optimization Based on Multi-Granularity Feature Selection and Model Fusion. Power Syst. Prot. Control.

[25] Mrutyunjaya S., Kishore D.P. (2018). Automatic Power Quality Events Recognition Based on Hilbert Huang Transform and Weighted Bidirectional Extreme Learning Machine. IEEE Trans. Ind. Inform..

[26] Elharrouss O., Himeur Y., Mahmood Y., Alrabaee S., Ouamane A., Bensaali F., Bechqito Y., Chouchane A. (2025). Leveraging Vision Transformers for Feature Extraction. Inf. Fusion.

[27] Chen Z., Tang X., Li Z., Zeng X. (2022). Type Identification and Time Location of Multiple Power Quality Disturbances Based on KF-ML-Aided DBN. IET Gener. Transm. Distrib..

[28] Chen J., Li Y., Wang Z. (2021). A BiLSTM-Attention Hybrid Model for Power Quality Disturbance Recognition. IEEE Trans. Ind. Inform..

[29] Zhang L., Wang X., Li Q. (2022). Data Augmentation for Power Quality Disturbance Classification Using GANs. Electr. Power Syst. Res..

[30] Kaveh H., Salarieh H., Hajiloo R. (2018). On the Control of Unknown Continuous Time Chaotic Systems by Applying Takens Embedding Theory. Chaos Solitons Fract..

[31] Kim H.S., Eykholt R., Salas J.D. (1999). Nonlinear Dynamics, Delay Times, and Embedding Windows. Physica D.

[32] Cao L.Y. (1997). Practical Method for Determining the Minimum Embedding Dimension of a Scalar Time Series. Physica D.

[33] Guo J., Si Z., Xiang J. (2023). Cycle Kurtosis Entropy Guided Symplectic Geometry Mode Decomposition for Detecting Faults in Rotating Machinery. ISA Trans..

[34] Zhang X.Y., Li C.S., Wang X.B. (2020). A Novel Fault Diagnosis Procedure Based on Improved Symplectic Geometry Mode Decomposition and Optimized SVM. Measurement.

[35] Shi L.L. (2016). Correlation Coefficient of Simplified Neuromorphic Sets for Bearing Fault Diagnosis. Shock Vib..

[36] IEEE (2019). IEEE Recommended Practice for Monitoring Electric Power Quality.

[37] Zhang X., Liu Z., Miao Q., Wang L. (2018). Bearing Fault Diagnosis Using a Whale Optimization Algorithm-Optimized Orthogonal Matching Pursuit with a Combined Time–Frequency Atom Dictionary. Mech. Syst. Signal Process..

[38] Bai L., Li W., Ren H., Li F., Yan T., Chen L. (2024). Weak Fault Feature Extraction of the Rotating Machinery Using Flexible Analytic Wavelet Transform and Nonlinear Quantum Permutation Entropy. Comput. Mater. Contin..

[39] Wang Z.Y., Yao L.G., Cai Y.W. (2020). Rolling Bearing Fault Diagnosis Using Generalized Refined Composite Multi-Scale Sample Entropy and Optimized Support Vector Machine. Measurement.

